# DFT Study of Molecular Structure, Electronic and Vibrational Spectra of Tetrapyrazinoporphyrazine, Its Perchlorinated Derivative and Their Al, Ga and In Complexes

**DOI:** 10.3390/ijms23105379

**Published:** 2022-05-11

**Authors:** Igor V. Ryzhov, Alexey V. Eroshin, Yuriy A. Zhabanov, Daniil N. Finogenov, Pavel A. Stuzhin

**Affiliations:** Research Institute of Chemistry of Macroheterocyclic Compounds, Ivanovo State University of Chemistry and Technology, Sheremetievskiy Av. 7, 153000 Ivanovo, Russia; ryzhoff.ihor@yandex.ru (I.V.R.); alexey.yeroshin@gmail.com (A.V.E.); dan.finogenof@gmail.com (D.N.F.); stuzhin@isuct.ru (P.A.S.)

**Keywords:** tetrapyrazinoporphyrazine, octachlorotetrapyrazinoporphyrazine, DFT study, molecular and electronic structure, electronic spectra, vibrational spectra

## Abstract

Electronic and geometric structures of metal-free, Al, Ga and In complexes with tetrapyrazinoporphyrazine (TPyzPA) and octachlorotetrapyrazinoporphyrazine (TPyzPACl_8_) were investigated by density functional theory (DFT) calculations and compared in order to study the effect of chlorination on the structure and properties of these macrocycles. The nature of the bonds between metal atoms and nitrogen atoms was described using the NBO-analysis. Simulation and interpretation of electronic spectra were performed with the use of time-dependent density functional theory (TDDFT). A description of calculated IR spectra was carried out based on the analysis of the distribution of the potential energy of normal vibrational coordinates.

## 1. Introduction

Tetrapyrrole macroheterocycles are essential for a wide range of technologies, including optoelectronics and information storage devices [1,2,3,4,5,6,7]. The complexes of tetrapyrazinoporphyrazine (H_2_TPyzPA) with metals possessing π-deficient heterocyclic fragments are promising compounds for organic electronics [8,9,10,11]. The results of the recent electrochemical study [8] of the perchlorinated complexes M(Cl)TPyzPACl_8_ (M = Al, Ga, In) allow them to be considered as potential acceptor materials. Recently this was also demonstrated for perchlorinated tripyrazinosubporphyrazines [12].

Fine tuning of the physico-chemical properties of the metal complexes with macroheterocyclic ligands requires a deep knowledge of their molecular and electronic structures. However, only a little attention has, of yet, been paid to structural investigations of the metal complexes of TPyzPA [13]. Solid-state X-ray studies of Fe(II) [14] and Sn(IV) complexes [15] revealed that the TPyzPACl_8_ macrocycle is nearly planar. The possible planarity of the structures of MTPyzPACl_8_ complexes gives rise to the extended π-electron conjugation, which in turn results in an increased packing density.

The present contribution is devoted to the combined computational investigation of the geometry structures, features of the metal–ligand bonding and spectral properties of M(Cl)TPyzPA (M = Al, Ga, In) complexes and their perchlorinated analogues. It also extends our recent study [16] of the tetrabenzoporphyrin (TBP) complexes with the same set of metals. Density functional theory (DFT) [17,18] was used as a theoretical framework since it was earlier established to provide a good description of the features of the geometry and electronic structures of the analogous macroheterocycles and their metal complexes [16,19,20,21,22,23,24,25].

## 2. Results

### 2.1. Molecular Structures

**Figure 1:**
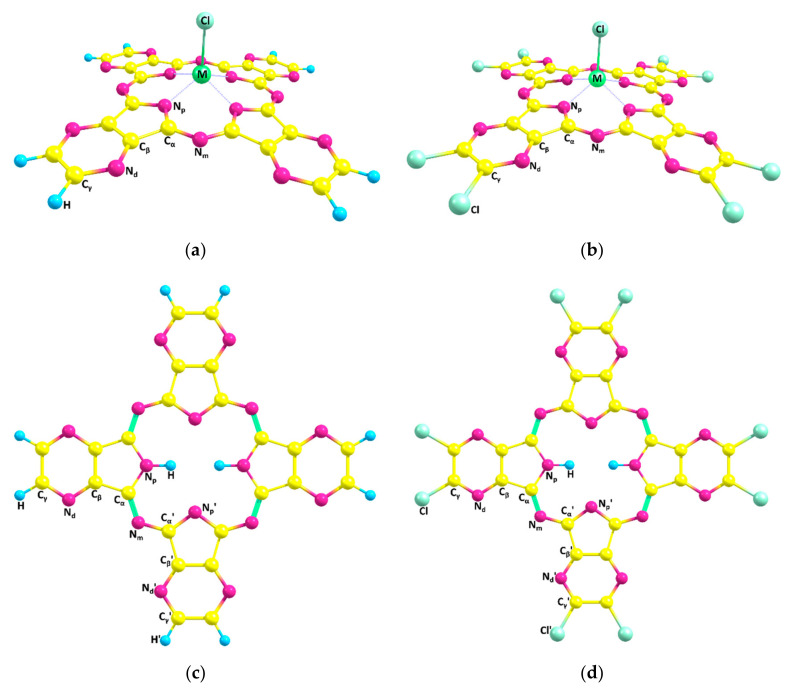
Molecular structures of the Al(III), Ga(III) and In(III) complexes with pyrazinoporphyrazine (M(Cl)TPyzPA) (**a**), octachloropyrazinoporphyrazines (M(Cl)TPyzPACl_8_) (**b**) and metal-free molecules ((**c**) and (**d**), respectively) with atom labeling.

According to the performed quantum chemical calculations, the equilibrium structures of the Al(III), Ga(III) and In(III) complexes of tetrapyrazinoporphyrazine (M(Cl)TPyzPA) and their perchlorinated analogues are doming-distorted and possess *C_4v_* symmetry (Figure 1). The distortion is caused by a metal atom as the metal-free H_2_TPyzPA and H_2_TPyzPACl_8_ molecules are planar (*D_2h_* symmetry). The H_2_TPyzPA internuclear distances obtained by the authors of [26] are systematically higher compared to our data. This discrepancy can be explained by the use of corrections for the dispersion interaction in this paper and pseudopotentials in [26]. The H_2_TPyzPA bond lengths calculated in [27] are also systematically higher than those obtained in this work. The main geometric parameters of compounds under consideration are given in Table 1.

The degree of the doming-distortion can be described by the distance M-X between the metal atom and the center of the plane formed by four nitrogen atoms N_p_ and the dihedral angle between the planes of opposite pyrrole rings α. The distance M-X increases in line with the size of a metal atom. Furthermore, the dihedral angle between the opposite pyrrole fragments α decreases by 10 degrees from Ga to In, while a slight change in this parameter is noted from Al to Ga (r_ionic_(Al) = 0.39, r_ionic_(Ga) = 0.47, r_ionic_(In) = 0.62) [28]. Structural parameters of the TPyzPA macrocyclic ligand are practically independent of the nature of a metal atom and peripheral –Cl substitution. The only noticeable change occurs for the peripheral C_γ_-C_γ_ bond that is 0.025 Å longer in the TPyzPACl_8_ metal complexes due to the electron-withdrawing effect of the –Cl substituents. A similar picture was previously observed for the complexes of tetrabenzoporphyrin with Al(Cl), Ga(Cl), In(Cl) [10] and porphyrazine, and tetrakis(1,2,5-thiadiazole)porphyrazine with Y(Cl), La(Cl) and Lu(Cl) [29]. The perimeter of the coordination cavity is not affected by the nature of a metal atom and increases by ca. 0.05 Å in the metal complexes as compared to the metal-free H_2_TPyzPA and H_2_TPyzPACl_8_; nevertheless, the distances between the nitrogen atoms of pyrrole fragments (N_p_…N_p_)_opp_ and (N_p_…N_p_)_adj_ are noticeably different (up to 0.2 Å). This can be explained by the fact that in the Al-Ga-In series, there is a consistent decrease in distances N_p_-C_α_ along with valence angles ∠(MN_p_C_α_) and an increase in C_α_-N_m_ distances and ∠(C_α_N_m_C_α_), which leads to the possibility of a significant difference in distances (N_p_…N_p_)_opp_ and (N_p_…N_p_)_adj_ without noticeable perimeter change.

### 2.2. NBO-Analysis

**Figure 2:**
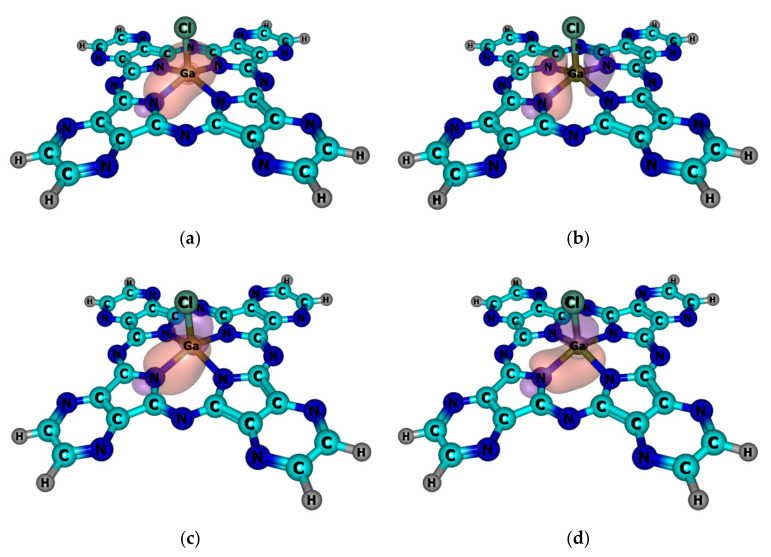
Schemes of the dominant donor–acceptor interactions between Ga and TPyzPA ligand. (**a**) The result of the orbital interaction of the type LP(N) → 4s(Ga) (E^(2)^ = 59.2 kcal mol^−1^); (**b**) the result of the orbital interaction of the type LP(N) → 4p_x_(Ga) (E^(2)^ = 30.7 kcal mol^−1^); (**c**) the result of the orbital interaction of the type LP(N) → 4p_y_(Ga) (E^(2)^ = 30.7 kcal mol^−1^); (**d**) the result of the orbital interaction of the type LP(N) → 4p_z_(Ga) (E^(2)^ = 14.3 kcal mol^−1^). Only one of the four corresponding interactions is demonstrated.

To gain a deeper insight into the electronic structures of M(Cl)TPyzPA and M(Cl)TPyzPACl_8_ complexes and features of the metal–ligand chemical bonding, we performed the NBO-analysis of the electron density distribution. The obtained results suggest that the chemical bonding between a metal atom and TPyzPACl_8_ macrocyclic ligand can be described in terms of the donor–acceptor interactions of the types: LP(N) → ns(M) and LP(N) → np(M), where n is the principal quantum number for the valence shell of the metal atom (n = 3 for Al, n = 4 for Ga, n = 5 for In). It should be noted that in the case of Al complexes, the interactions occur between LP(N) and two 3p(Al) orbitals, while for the complexes of Ga and In, all three valence p-orbitals provide a favorable overlap (Figure 2).

Comparing periphery-substituted TPyzPACl_8_ complexes with their non-substituted analogues (Table 2), we found that peripheral –Cl substituents do not affect the characteristics of the metal–ligand bonding. The enhanced covalent contribution into the Ga–N bond is in line with the trend of the electronegativities of the metal atoms: Al (χ = 1.47), Ga (χ = 1.82) and In (χ = 1.49) [30,31]. According to Table 2, the NPA charge of Al is the most positive, in contrast to the NPA charge of Ga. More than that, an analogical situation is observed for the negativity of NPA charges of chlorine atoms. Therefore, these charges also correlate with the electronegativities of the metals.

### 2.3. Electronic Absorption Spectra

**Figure 3:**
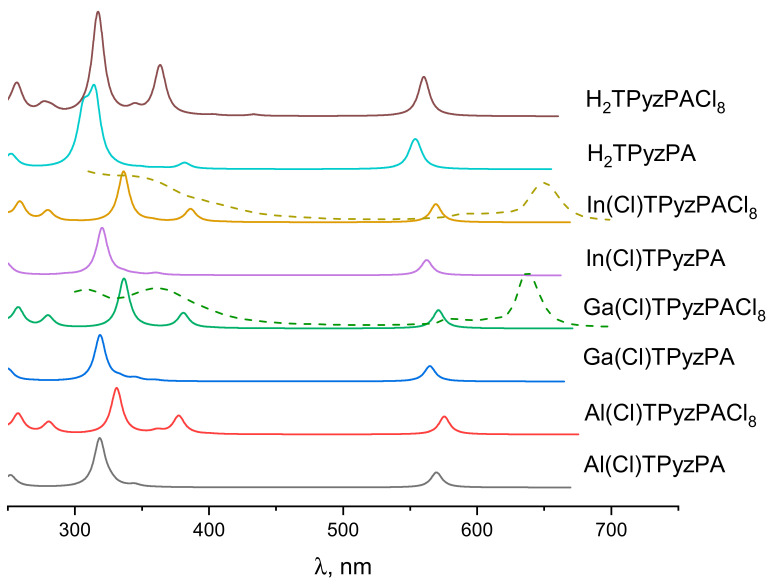
Simulated (solid lines) and experimental (dashed lines) electronic absorption spectra for M(Cl)TpyzPA and M(Cl)TPyzPACl_8_ (M = Al, Ga, In) and its metal-free complexes.

Analyzing the absorption spectra (Figure 3), we can note that three intense absorption bands are observed in the visible light and near UV in the case of M(Cl)TPyzPACl_8_, in contrast to M(Cl)TPyzPA, of which spectra contain only two bands in the visible light and near UV range. The B_1_-band in the latter has low oscillator strength, so it is practically imperceptible in the absorption spectrum, while this peak is clearly observed in the perchlorinated complexes. This can be related to the fact that the peripheral Cl atoms contribute significantly to 6a_1_ molecular orbital (MO) in M(Cl)TPyzPACl_8_ in contrast to the hydrogen atoms in M(Cl)TPyzPA to 3a_1_ MO (Figure S1). The B_1_-band is predominantly formed by the electron transitions from these orbitals to doubly-degenerated LUMOs (Table 3).

It can be seen that the position of the Q-band maxima in the electronic absorption spectra shifted bathochromically in the series Ga < Al < In. The changes in the theoretical and experimental spectra are similar to each other and therefore allow the nature of the results obtained to be explained. This indicates that the HOMO–LUMO gap is influenced not only by the degree of doming-distortion of the macrocycle, but also by electronegativity of the central metal, which is the highest for Ga. In addition, a minor bathochromic shift of the Q-band occurs for perchlorinated complexes M(Cl)TPyzPACl_8_ as compared to M(Cl)TPyzPA. Noteworthy is the single absorption maximum (B-band) in the near UV Soret region (300–420 nm) that appears for M(Cl)TPyzPA, while B_1_- and B_2_-bands are present for M(Cl)TPyzPACl_8_. Experimental spectra of Ga(OH)TPyzPACl_8_ and In(OH)TPyzPACl_8_ can be found in ESI (Figures S2 and S3).

In addition, attention should be paid to the pronounced hyperchromic effect in metal-free complexes caused by the superposition of several absorption bands with close wavelengths. The composition of the excited states forming the absorption bands is given in Table 3.

**Figure 4:**
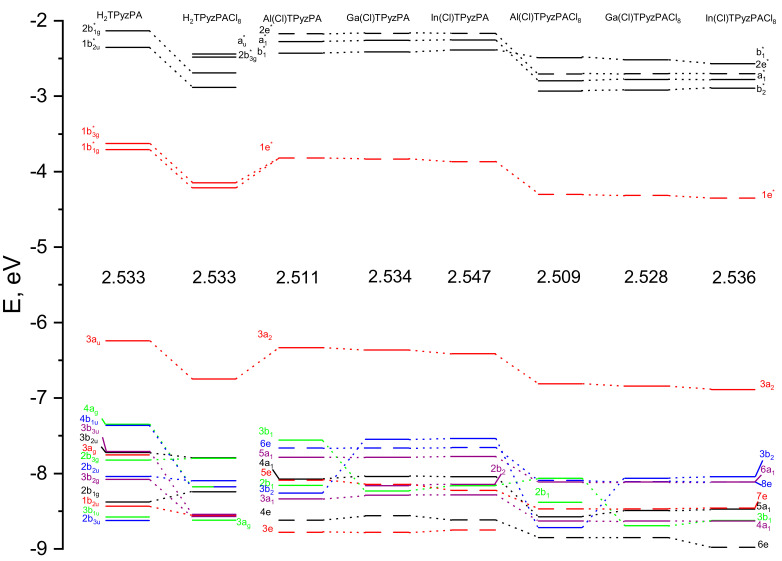
Molecular orbitals (MO) level diagram for H_2_TPyzPA, H_2_TPyzPACl_8_, M(Cl)TPyzPA and M(Cl)TPyzPACl_8_ complexes. The values of higher occupied molecular orbital–lowest unoccupied molecular orbital (HOMO–LUMO) gaps are given in eV.

It can be seen that the excited states of the compounds under consideration are formed as a result of similar sets of electron transitions. The main composition of the excited states responsible for the Q-band consists of transitions between the frontier π-orbitals. Hence Q-band wavelengths correlate with the HOMO–LUMO gaps. The molecular orbitals level diagram is shown in Figure 4. Stabilization of LUMO upon peripheral chlorination of the TPyzPA macrocycle correlates with the electrochemical data [8], indicating that the reduction potentials for Cl_8_TPyzPA complexes are shifted by 0.4–0.5 V in a less negative region as compared to the values typical for unsubstituted TPyzPA complexes [10]. It is also worth noting that the 1^1^E states are formed mainly by Gouterman-type transitions *a*_2_ → e* for metal complexes and transitions of the *a_u_*→*b*_1*g*_ and *a_u_*→*b*_3*g*_ types for metal-free ones due to the lower symmetry of the latter [32,33,34]. The situation is typical for a number of macrocycles of similar structure [35,36,37]. Despite the fact that the higher energy states are formed by almost the same electronic transitions, they have a different composition, and therefore energy.

Considering the shapes of the molecular orbitals (MOs) involved in the most probable electronic transitions, we can conclude that the metal nature should not affect the Q-band position since the boundary MOs are the linear combination of atomic orbitals (LCAO) of the macrocyclic ligand. Nevertheless, the size of the coordination cavity depends on the metal nature: changing the geometric structure provides an indirect effect on the position of the Q-band. Moreover, the shape of the LUMOs depends on the metal nature (Figure S1). In the case of Al, it consists of the bonding π-orbitals lying along the C_α_-C_β_ bond and antibonding π-orbitals located around the C_α_-N_m_ and C_α_-N_p_ bonds, while for Ga and In, the C_α_-N_p_ bonds have a significant contribution to the bonding π-orbital, probably due to the influence of the d-sublevel of the metal. The HOMO consists of the AOs localized on carbon atoms of pyrrolic fragments, which is a typical picture for porphyrazines [10,19,25,38]. Note that peripheral Cl atoms contribute to the formation of the MOs involved in the electronic transitions corresponding to the B_1_-band in the absorption spectrum. For the rest, the MO’s form is similar for all compounds; therefore, it can be concluded that the ligand rather than metal has a decisive influence on the absorption spectrum.

### 2.4. IR Spectra

The IR spectra were simulated on the basis of the normal mode frequencies and band intensities, which have been calculated by the DFT (PBE0/def2-TZVP) method in a harmonic approximation.

Bands of weak intensity appear in the region up to 600 cm^−1^, as well as the shift of bands in the range from 600 to 1000 cm^−1^ occurs with the substitution of hydrogen atoms by the metal. It should be noted that the most intensive vibrational transitions of the metal complexes are degenerate, while the splitting of the bands in the case of the metal-free H_2_TPyzPA and H_2_TPyzPACl_8_ is observed due to the lower symmetry. It results in two peaks with close frequencies formed by the normal vibrations of the pyrrole and pyrrolenine fragments. A medium peak of the N_p_-H stretching at 3552 cm^−1^ and 3554 cm^−1^ (H_2_TPyzPA and H_2_TPyzPACl_8_, respectively) is typical for macrocycles such as porphyrazines [25]. According to the experimental data [8,26], these frequencies are 3286 and 3287 cm^−1^, respectively.

In-plane vibrations contribute to the medium band at 1400 cm^−1^ in the spectra of M(Cl)TPyzPA. Noteworthy are the main differences in the M(Cl)TPyzPA IR spectra observed in the range of 300–600 cm^−1^ related to the weak bands with a strong contribution of the M-Cl stretching.

It is worth noting that a low-frequency band shift occurs in both M(Cl)TPyzPA and M(Cl)TPyzPACl_8_ in the Al→Ga→In series. Among the most intense peaks, the only exception is the ω_92_-ω_93_ band at the ~1140 cm^−1^ for MTPyzPA, which corresponds to in-plane vibrations of the macrocyclic core. Moreover, a significant decrease in these bands’ intensity occurs with an increase in the ionic radius of a metal [28].

**Figure 5:**
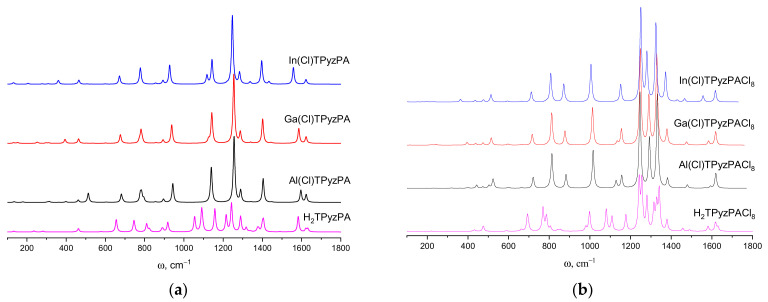
Calculated IR spectra of M(Cl)TPyzPA (**a**) and M(Cl)TPyzPACl_8_ (**b**).

The IR spectra of M(Cl)TPyzPA are similar in the range of 600–3500 cm^−1^ where the influence of the metal is almost absent. The replacement of H by Cl leads to the vanishing of the bands at 3190 cm^−1^, corresponding to the C-H stretching vibrations, and an increase in the relative intensity of the peaks in the 800–1300 cm^−1^ region. Simulated spectra are shown in Figure 5. The most intense bands at ~1250 cm^−1^ of all investigated compounds are predominantly stretching vibrations of the bonds of pyrazine rings. Unlike M(Cl)TPyzPA spectra with one peak with a relative intensity >50%, the M(Cl)TPyzPACl_8_ spectra also contain the strong bands ω_117_-ω_118_, composed by N_d_-C_γ_ and C_β_-N_d_ stretching vibrations. Moreover, M(Cl)TPyzPACl_8_ spectra include more relatively intense peaks compared to MTPyzPA. A description of the main vibrations is listed in Table 4 (full list of the most active vibrations can be found in Supplementary Materials, Table S1). It is important to mention that the calculated spectra of Ga(Cl)TPyzPACl_8_ and In(Cl)TPyzPACl_8_ are consistent with the experimental ones with a factor ~0.95. Actually, the main differences may be caused by another axial ligand (OH instead of Cl). Experimental spectra can be found in ESI (Figures S4 and S5).

## 3. Materials and Methods

### 3.1. Synthesis

Octachlorotetrapyrazinoporphyrazinatogallium(III) hydroxide, [Ga(OH)TPyzPACl_8_]. A mixture of 5,6-dichloropyrazine-2,3-dicarbonitrile (200 mg, 1 mmol) and gallium(III) hydroxydiacetate (50 mg, 0.24 mmol) was melted at 200 °C for 10 min. The obtained solid was powdered, washed with CH_2_Cl_2_, then dissolved in conc. H_2_SO_4_, precipitated by pouring into ice-water, centrifuged and washed with MeOH. After drying, the complex was obtained as dark green hydrated material.

Octachlorotetrapyrazinoporphyrazinatoindium(III) hydroxide, [In(OH)TPyzPACl_8_]. A mixture of 5,6-dichloropyrazine-2,3-dicarbonitrile (1) (200 mg, 1 mmol) and indium(III) hydroxydiacetate (50 mg, 0.2 mmol) was melted at 200 °C for 10 min. The obtained solid was powdered, washed with CH_2_Cl_2_, then dissolved in THF and chromatographed on silica with THF as eluent. The eluted product was precipitated by pouring into water, centrifuged and washed with MeOH. After drying, the complex was obtained as dark green hydrated material [8].

The IR spectra were measured on an IR-spectrometer Cary 630 FT-IR using KBr pellets. UV–Vis spectra were recorded using a Cary UV-Vis spectrophotometer in THF solution and can be found in ESI.

### 3.2. Computational Details

The DFT study of M(Cl)TPyzPA and M(Cl)TPyzPACl_8_ included geometry optimization and calculations of the harmonic vibrations, followed by calculation of the electronic absorption spectrum by the TDDFT method. The number of the calculated excited states was 30. The calculations were performed using the PBE0 functional with the density functional dispersion correction D3 provided by Grimme [40] with the def2-TZVP basis set [41] taken from the EMSL BSE library [42,43,44]. Firefly QC package [45], which is partially based on the GAMESS (US) [46] source code, was used to obtain the optimized geometry, electronic absorption spectra and NBO-analysis. The optimized Cartesian coordinates of H_2_TPyzPA, H_2_TPyzPACl_8_ and their metal complexes with Al, Ga, In are available in the Supplementary Materials.

The calculations of IR spectra were carried out with use of Gaussian09 [47] software package due to the fact it applies analytical functions in the second derivatives computing. The molecular models and orbitals demonstrated in the paper were visualized by means of the Chemcraft program [48].

## 4. Conclusions

The geometry and electronic structure of TPyzPA were investigated using PBE0-D3 functional with basis set def2-TZVP. The distance M-X between the metal atom and the center of the plane formed by four nitrogen atoms N_p_ increases in line with the size of a metal atom. Structural parameters of the TPyzPA macrocyclic ligand are practically independent of the nature of a metal atom and peripheral −Cl substitution. The fact that (N_p_…N_p_)_opp_ and (N_p_…N_p_)_adj_ are substantially different for Al, Ga and In complexes, while the perimeter of the coordination cavity almost remains invariant for both M(Cl)TPyzPA and M(Cl)TPyzPACl_8_ complexes, indicates that the degree of distortion increases from Al to In. At the same time, the substitution of H-atoms for Cl mainly affects the internuclear distances of pyrazine rings and has a negligible effect on the N_p_-C_α_ and C_α_-N_m_.

According to the NBO-analysis of electron density distribution, the strong donor–acceptor interactions of the types: LP(N) → ns(M) and LP(N) → np(M), where n is the principal quantum number for the valence shell of the metal atom, stabilize the complexes.

It was found that the HOMO–LUMO gap is slightly affected by the nature of the metal and increases in the Al-Ga-In series. The substitution of H-atoms for Cl leads to a decrease in the energy of the frontier MOs of M(Cl)TPyzPACl_8_ as compared to M(Cl)TPyzPA. Furthermore, the LUMO is stabilized much more than the HOMO, resulting in a smaller HOMO–LUMO gap.

Simulated electronic absorption spectra show that the metal nature slightly influences the position of the band’s position. In the series Al→Ga→In→H_2_, the Q-band is shifted to a shorter wavelength. In addition, a minor bathochromic shift occurs in M(Cl)TPyzPA compared to M(Cl)TPyzPACl_8_. The excited states corresponding to the Q-bands are composed mainly of Gouterman-type transitions. The electronic absorption spectra of TPyzPACl_8_ contain three intense maxima, while only two peaks were found for TPyzPA. The additional B_1_ band in the M(Cl)TPyzPACl_8_ spectra is caused by the transitions from MOs with a contribution of AOs of the peripheral Cl atoms.

The calculated IR spectra of TPyzPACl_8_ have a significant difference in comparison with TPyzPA due to heavy Cl atoms being involved in the vibrations that form medium and strong bands in the region of 1000–1500 cm^−1^.

## Figures and Tables

**Table 1:** Internuclear distances (r_e_, in Å) and valence angles (∠, in deg.) of the equilibrium structures by PBE0-D3/def2-TZVP calculations.

	Al(Cl)TPyzPA	Ga(Cl)TPyzPA	In(Cl)TPyzPA	H_2_TPyzPA
Symmetry	*C_4v_*	*C_4v_*	*C_4v_*	*D*_2h_
Distances				
M-N_p_/M-N_p_′	1.981	2.022	2.161	-
M-Cl_ax_	2.145	2.187	2.336	-
N_p_-C_α_/N_p_′-C_α_′	1.371	1.367	1.364	1.368/1.355
C_α_-N_m_/C_α_′-N_m_	1.308	1.310	1.316	1.304/1.321
C_α_-C_β_/C_α_′-C_β_′	1.450	1.453	1.458	1.450/1.464
C_β_-C_β_/C_β_′-C_β_′	1.391	1.393	1.399	1.401/1.393
C_β_-N_d_/C_β_′-N_d_′	1.328	1.328	1.326	1.329/1.324
N_d_-C_γ_/N_d_′-C_γ_′	1.322	1.322	1.323	1.320/1.327
C_γ_-C_γ_/C_γ_′-C_γ_′	1.408	1.407	1.405	1.408/1.401
C_γ_-H/C_γ_′-H’	1.086	1.086	1.086	1.086/1.086
(N_p_…N_p_)_opp_	3.859	3.918	4.037	4.054/3.899
(N_p_…N_p_)_adj_	2.728	2.770	2.854	2.812
P ^1^	21.432	21.416	21.440	21.376
Bond angles				
∠(Cl_ax_MN_p_)	103.1	104.3	110.9	-
∠(MN_p_C_α_)	125.2	124.5	123.6	123.2
∠(N_p_C_α_N_m_)/(N_p_′C_α_′N_m_)	127.9	128.0	128.0	128.5/128.1
∠(C_α_N_m_C_α_′)	122.5	123.4	125.5	124.2
∠(C_α_C_β_N_d_)/(C_α_′C_β_′N_d_′)	129.8	129.8	129.8	129.0/130.6
∠(C_β_N_d_C_γ_)/(C_β_′N_d_′C_γ_′)	113.2	113.3	113.6	113.6/113.3
M-X ^2^	0.448	0.499	0.772	-
α ^3^	175.5	174.3	164.7	180
	Al(Cl)TPyzPACl_8_	Ga(Cl)TPyzPACl_8_	In(Cl)TPyzPACl_8_	H_2_TPyzPACl_8_
Symmetry	*C_4v_*	*C_4v_*	*C_4v_*	*D*_2h_
Distances				
M-N_p_/M-N_p_′	1.980	2.021	2.162	-
M-Cl_ax_	2.142	2.183	2.332	-
N_p_-C_α_/N_p_′-C_α_′	1.372	1.368	1.364	1.368/1.356
C_α_-N_m_/C_α_′-N_m_	1.308	1.310	1.316	1.304/1.321
C_α_-C_β_/C_α_′-C_β_′	1.447	1.450	1.455	1.448/1.461
C_β_-C_β_/C_β_′-C_β_′	1.384	1.387	1.392	1.394/1.386
C_β_-N_d_/C_β_′-N_d_′	1.330	1.329	1.328	1.330/1.325
N_d_-C_γ_/N_d_′-C_γ_′	1.305	1.306	1.307	1.304/1.310
C_γ_-C_γ_/C_γ_′-C_γ_′	1.433	1.432	1.430	1.433/1.424
C_γ_-Cl/C_γ_′-Cl′	1.709	1.709	1.709	1.709/1.712
(N_p_…N_p_)_opp_	3.856	3.915	4.031	4.051/3.892
(N_p_…N_p_)_adj_	2.726	2.768	2.850	2.809
P ^1^	21.440	21.424	21.440	21.376
Bond angles				
∠(Cl_ax_MN_p_)	103.2	104.5	111.2	-
∠(MN_p_C_α_)	125.2	124.5	123.5	123.3
∠(N_p_C_α_N_m_)/(N_p_′C_α_′N_m_)	128.0	128.1	128.1	128.5/128.2
∠(C_α_N_m_C_α_′)	122.3	123.3	125.3	124.0
∠(C_α_C_β_N_d_)/(C_α_′C_β_′N_d_′)	130.3	130.3	130.3	129.5/131.1
∠(C_β_N_d_C_γ_)/(C_β_′N_d_′C_γ_′)	114.9	115.0	115.2	115.3/114.9
M-X ^2^	0.451	0.504	0.782	-
A ^3^	175.3	174.0	164.6	180

^1^ P is the coordination cavity perimeter (in Å). ^2^ X is dummy atom located in center between N_p_ atoms. ^3^ α is the dihedral angle between planes of opposite pyrrole rings.

**Table 2:** Selected parameters of M(Cl)PyzPA complexes from NBO calculations.

	Al(Cl) TPyzPA	Al(Cl) TPyzPACl_8_	Ga(Cl) TPyzPA	Ga(Cl) TPyzPACl_8_	In(Cl) TPyzPA	In(Cl) TPyzPACl_8_
*E*(HOMO),eV	−6.329	−6.803	−6.362	−6.836	−6.411	−6.882
*E*(LUMO),eV	−3.815	−4.294	−3.829	−4.308	−3.864	−4.343
∆*E*, eV	2.514	2.509	2.533	2.528	2.547	2.539
*q*(M) NPA, *e*	1.718	1.716	1.637	1.635	1.694	1.692
*q*(N) NPA, *e*	−0.651	−0.651	−0.631	−0.631	−0.623	−0.623
*q*(Cl) NPA, *e*	−0.550	−0.545	−0.523	−0.518	−0.528	−0.521
configuration	3s^0.42^3p^0.83^	3s^0.42^3p^0.83^	4s^0.53^4p^0.81^	4s^0.53^4p^0.81^	5s^0.54^5p^0.76^	5s^0.54^5p^0.76^
∑ *E*(d-a), kcal mol^−1^	526.0	526.5	539.6	539.0	510.7	507.8
*Q*(M-N), *e*	0.335	0.330	0.343	0.342	0.327	0.325
*r*(M-N), Å	1.981	1.980	2.022	2.021	2.161	2.162

**Table 3:** Calculated composition of the lowest excited states and corresponding oscillator strengths for TPyzPA and TPyzPzCl_8_ complexes.

Al(Cl)TPyzPA					Al(Cl)TPyzPACl_8_				
State	Composition (%)	λ, nm	*f*	exp. λ, nm	State	Composition (%)	λ, nm	*f*	exp. λ, nm
1^1^E	$4a_{1}\to 1e^{*}$(8) $3a_{2}\to 1e^{*}$(90)	570	0.31		1^1^E	$5a_{1}\to 1e^{*}$(5) $3a_{2}\to 1e^{*}$(90)	576	0.37	643
7^1^E	$3a_{1}\to 1e^{*}$(54) $2b_{2}\to 1e^{*}$(17) $4a_{1}\to 1e^{*}$(22)	327	0.07		4^1^E	$2b_{1}\to 1e^{*}$(5) $6a_{1}\to 1e^{*}$(77) $3b_{1}\to 1e^{*}$(7) $3a_{2}\to 2e^{*}$(5)	377	0.37	
9^1^E	$3a_{1}\to 1e^{*}$(27) $4a_{1}\to 1e^{*}$(58) $3a_{2}\to 1e^{*}$(6) $3a_{2}\to 2e^{*}$(6)	318	1.00		8^1^E	$5a_{1}\to 1e^{*}$(70) $6a_{1}\to 1e^{*}$(7) $3a_{2}\to 1e^{*}$(7) $3a_{2}\to 2e^{*}$(9)	331	0.95	363
Ga(Cl)TPyzPA					Ga(Cl)TPyzPACl_8_				
State	Composition (%)	λ, nm	*f*	exp. λ, nm	State	Composition (%)	λ, nm	*f*	exp. λ, nm
1^1^E	$4a_{1}\to 1e^{*}$(8) $3a_{2}\to 1e^{*}$(90)	565	0.32		1^1^E	$5a_{1}\to 1e^{*}$(6) $3a_{2}\to 1e^{*}$(90)	571	0.38	638
7^1^E	$3a_{1}\to 1e^{*}$(26) $2b_{2}\to 1e^{*}$(34) $4a_{1}\to 1e^{*}$(32)	333	0.05		4^1^E	$5a_{1}\to 1e^{*}$(6) $6a_{1}\to 1e^{*}$(75) $4b_{2}\to 1e^{*}$(13)	381	0.31	
9^1^E	$3a_{1}\to 1e^{*}$(47) $4a_{1}\to 1e^{*}$(38) $3a_{2}\to 1e^{*}$(6) $3a_{2}\to 2e^{*}$(5)	319	0.95		8^1^E	$5a_{1}\to 1e^{*}$(63) $6a_{1}\to 1e^{*}$(8) $3a_{2}\to 1e^{*}$(8) $3a_{2}\to 2e^{*}$(16)	337	1.03	360
In(Cl)TPyzPA					In(Cl)TPyzPACl_8_				
State	Composition (%)	λ, nm	*f*	exp. λ, nm	State	Composition (%)	λ, nm	*f*	exp. λ, nm
1^1^E	$4a_{1}\to 1e^{*}$(8) $3a_{2}\to 1e^{*}$(89)	562	0.32	589	1^1^E	$3a_{2}\to 1e^{*}$(90) $5a_{1}\to 1e^{*}$(6)	569	0.38	649
7^1^E	$3a_{1}\to 1e^{*}$(36) $2b_{2}\to 1e^{*}$(26) $4a_{1}\to 1e^{*}$(28) $3a_{2}\to 2e^{*}$(7)	336	0.04	335	4^1^E	$6a_{1}\to 1e^{*}$(76) $5a_{1}\to 1e^{*}$(6) $3b_{2}\to 1e^{*}$(14)	386	0.26	
9^1^E	$3a_{1}\to 1e^{*}$(43) $4a_{1}\to 1e^{*}$(39) $3a_{2}\to 1e^{*}$(7) $3a_{2}\to 2e^{*}$(7)	320	0.98		8^1^E	$5a_{1}\to 1e^{*}$(55) $6a_{1}\to 1e^{*}$(6) $3a_{2}\to 1e^{*}$(8) $3a_{2}\to 2e^{*}$(22)	336	1.05	339
H_2_TPyzPA					H_2_ TPyzPACl_8_				
State	Composition (%)	λ, nm	*f*	exp. λ, nm	State	Composition (%)	λ, nm	*f*	exp. λ, nm
1^1^B_1u_	$3a_{u}\to 1b_{1g}^{*}$(90)	555	0.35	645	1^1^B_1u_	$3a_{u}\to 1b_{1g}^{*}$(90)	560	0.43	656
1^1^B_3u_	$3a_{u}\to 1b_{3g}^{*}$(86) $2b_{2u}\to 1b_{1g}^{*}$(5) $3b_{2u}\to 1b_{1g}^{*}$(8)	552	0.33	611	1^1^B_3u_	$1b_{2u}\to 1b_{1g}^{*}$(5) $2b_{2u}\to 1b_{1g}^{*}$(7) $3a_{u}\to 1b_{3g}^{*}$(86)	560	0.39	626
2^1^B_3u_	$2b_{2u}\to 1b_{1g}^{*}$(12) $3b_{2u}\to 1b_{1g}^{*}$(80)	382	0.12		3^1^B_3u_	$1b_{2u}\to 1b_{1g}^{*}$(5) $2b_{2u}\to 1b_{1g}^{*}$(85) $3a_{u}\to 2b_{3g}^{*}$(5)	364	0.78	358
4^1^B_3u_	$1b_{2u}\to 1b_{1g}^{*}$(7) $2b_{2u}\to 1b_{1g}^{*}$(66) $3b_{2u}\to 1b_{1g}^{*}$(9) $3a_{u}\to 1b_{3g}^{*}$(9) $3a_{u}\to 2b_{3g}^{*}$(6)	315	1.32	330	4^1^B_1u_	$1b_{2u}\to 1b_{3g}^{*}$(18) $2b_{2u}\to 1b_{3g}^{*}$(78)	362	0.26	358
4^1^B_1u_	$1b_{2u}\to 1b_{3g}^{*}$(62) $2b_{2u}\to 1b_{3g}^{*}$(33)	311	0.29		4^1^B_3u_	$1b_{2u}\to 1b_{1g}^{*}$(6) $3a_{u}\to 2b_{3g}^{*}$(91)	345	0.13	
5^1^B_1u_	$1b_{2u}\to 1b_{3g}^{*}$(33) $2b_{2u}\to 1b_{3g}^{*}$(42) $3b_{2u}\to 1b_{3g}^{*}$(10) $3a_{u}\to 1b_{1g}^{*}$(6) $3a_{u}\to 2b_{1g}^{*}$(5)	306	0.94		5^1^B_3u_	$1b_{2u}\to 1b_{1g}^{*}$(79) $3a_{u}\to 1b_{3g}^{*}$(8)	319	0.88	
					5^1^B_1u_	$1b_{2u}\to 1b_{3g}^{*}$(68) $2b_{2u}\to 1b_{3g}^{*}$(12)	316	1.34	

**Table 4:** Assignment of the IR vibrations of the M(Cl)TPyzPA and M(Cl)TPyzPACl_8_ complexes.

Frequency, cm^−1^	I_rel_, %	Symmetry	Assignment ^1^	Exp, cm^−1^
H_2_TPyzPA				KBr [26]
1091 (ω_86_)	78	B_1u_	r(N_p_-C_α_) (29), r(N_m_-C_α_) (18), r(N_d_-C_γ_) (10), r(C_γ_-C_γ_) (12)	1040
1157 (ω_90_)	81	B_2u_	r(N_p_-C_α_) (23), r(N_m_-C_α_) (18), r(C_α_-C_β_) (6), r(C_β_-N_d_) (8), r(N_d_-C_γ_) (6), φ(C_α_-N_p_-H_c_) (8), φ(C_β_-N_d_-C_γ_) (7)	1121
1242 (ω_95_)	100	B_1u_	r(C_α_-C_β_) (14), r(C_β_-C_β_) (10), r(N_d_-C_γ_) (10), φ(N_d_-C_γ_-H_s_) (7)r(C_β_-N_d_) (6), r(C_β_-C_β_) (42), r(C_γ_-Cl) (26), φ(N_d_-C_γ_-Cl) (6)	1198
3193 (ω_141_)	24	B_2u_	r(H_s_-C_γ_) (99)	3051
3552 (ω_143_)	45	B_1u_	r(H_c_-N_p_) (99)	3286
H_2_TPyzPACl_8_				KBr [8]
1242 (ω_107_)	100	B_1u_	r(C_β_-N_d_) (6), r(C_β_-C_β_) (42), r(C_γ_-Cl) (26), φ(N_d_-C_γ_-Cl) (6)	1192
1255 (ω_109_)	98	B_2u_	r(C_β_-N_d_) (8), r(C_γ_-C_γ_) (46), r(C_γ_-Cl) (21), φ(N_d_-C_γ_-Cl) (6)	1235
1281 (ω_111_)	67	B_1u_	r(C_α_-C_β_) (8), r(C_β_-C_β_) (19), r(N_d_-C_γ_) (13), r(C_γ_-C_γ_) (13)	
1328 (ω_116_)	52	B_1u_	r(N_p_-C_α_) (7), r(N_m_-C_α_) (11), r(C_β_-N_d_) (12), r(N_d_-C_γ_) (45)	
1340 (ω_117_)	83	B_2u_	r(N_p_-C_α_) (8), r(C_α_-C_β_) (10), r(C_β_-C_β_) (8), r(C_β_-N_d_) (19), φ(C_α_-N_p_-H_c_) (28)	
3554 (ω_143_)	27	B_1u_	r(H_c_-N_p_) (99)	3287
Al(Cl)TPyzPA				
1140 (ω_92_-ω_93_)	53	E	r(N_p_-C_α_) (24), r(N_m_-C_α_) (14), r(C_α_-C_β_) (10), r(C_β_-N_d_) (12), r(N_d_-C_γ_) (9)	
1255 (ω_98_-ω_99_)	100	E	r(N_p_-C_α_) (5), r(C_α_-C_β_) (15), r(C_β_-C_β_) (14), r(N_d_-C_γ_) (20), φ(C_β_-N_d_-C_γ_) (9)	
1404(ω_113_-ω_114_)	36	E	r(C_β_-C_β_) (12), r(C_γ_-C_γ_) (13), φ(N_d_-C_γ_-H_s_) (36), φ(C_γ_-C_γ_-H_s_) (23)	
3189 (ω_142_-ω_143_)	18	E	r(H_s_-N_p_) (99)	
Al(Cl)TPyzPACl_8_				KBr [8]
1246 (ω_111_-ω_112_)	100	E	r(C_γ_-C_γ_) (43), r(C_γ_-Cl) (25), φ(N_d_-C_γ_-Cl) (6)	1168
1293 (ω_115_-ω_116_)	52	E	r(N_p_-C_α_) (8), r(C_β_-C_β_) (15), r(N_d_-C_γ_) (25), r(C_γ_-C_γ_) (13)	1260
1331 (ω_117_-ω_118_)	97	E	r(C_α_-C_β_) (8), r(C_β_-N_d_) (23), r(N_d_-C_γ_) (49)	1323
Ga(Cl)TPyzPA				
1142 (ω_92_-ω_93_)	44	E	r(N_p_-C_α_) (49), r(N_m_-C_α_) (11), r(C_α_-C_β_) (9), r(N_d_-C_γ_) (9)	
1254 (ω_98_-ω_99_)	100	E	r(N_p_-C_α_) (6), r(C_α_-C_β_) (12), r(C_β_-C_β_) (11), r(C_β_-N_d_) (9), r(N_d_-C_γ_) (18)	
1401 (ω_113_-ω_114_)	35	E	r(C_β_-C_β_) (11), r(C_γ_-C_γ_) (12), φ(N_d_-C_γ_-H_s_) (36), φ(C_γ_-C_γ_-H_s_) (23)	
3194 (ω_142_-ω_143_)	17	E	r(H_s_-N_p_) (99)	
Ga(Cl)TPyzPACl_8_				this work
1248 (ω_111_-ω_112_)	100	E	r(C_γ_-C_γ_) (41), r(C_γ_-Cl) (24), φ(N_d_-C_γ_-Cl) (6)	1232
1290 (ω_115_-ω_116_)	50	E	r(N_p_-C_α_) (6), r(C_α_-C_β_) (7), r(C_β_-C_β_) (17), r(N_d_-C_γ_) (26), r(C_γ_-C_γ_) (13)	
1328 (ω_117_-ω_118_)	94	E	r(C_α_-C_β_) (8), r(C_β_-N_d_) (22), r(N_d_-C_γ_) (50)	1349
In(Cl)TPyzPA				KBr [39]
1143 (ω_92_-ω_93_)	36	E	r(N_p_-C_α_) (49), r(N_m_-C_α_) (10), r(C_α_-C_β_) (6), r(N_d_-C_γ_) (10)	1100
1247 (ω_98_-ω_99_)	100	E	r(N_p_-C_α_) (5), r(C_α_-C_β_) (15), r(C_β_-C_β_) (14), r(N_d_-C_γ_) (20)	1213
1396 (ω_113_-ω_114_)	34	E	r(C_β_-C_β_) (10), r(C_γ_-C_γ_) (10), φ(N_d_-C_γ_-H_s_) (34), φ(C_γ_-C_γ_-H_s_) (22)	1364
3194 (ω_142_-ω_143_)	17	E	r(H_s_-N_p_) (99)	3316
In(Cl)TPyzPACl_8_				this work
1250 (ω_111_-ω_112_)	100	E	r(C_γ_-C_γ_) (44), r(C_γ_-Cl) (26), φ(N_d_-C_γ_-Cl) (6)	1264
1280 (ω_114_-ω_115_)	50	E	r(N_p_-C_α_) (5), r(C_α_-C_β_) (7), r(C_β_-C_β_) (19), r(N_d_-C_γ_) (26)	1323
1325 (ω_117_-ω_118_)	83	E	r(C_α_-C_β_) (8), r(C_β_-N_d_) (23), r(N_d_-C_γ_) (49)	1364

^1^ Coordinates are listed provided that their contributions (shown in parentheses) are greater than ~5%. Assignment of vibrational modes based on potential energy distribution. The following designations of the coordinates are used: r—stretching of the bond; φ—bending, a change in the angle; OPB—out-of-plane bending; θ—a change in the dihedral angle. Experimental frequencies are given for metal complexes with an axial–OH ligand.

## Data Availability

The data presented in this study are available on request from the corresponding author.

## Supplementary Materials

The following supporting information can be downloaded at: www.mdpi.com/article/10.3390/ijms23105379/s1.
