# Supplementary material for: DFT Study of Molecular Structure, Electronic and Vibrational Spectra of Tetrapyrazinoporphyrazine, Its Perchlorinated Derivative and Their Al, Ga and In Complexes
Source: Int J Mol Sci. 2022 May 11;23(10):5379. doi: 10.3390/ijms23105379 (PMC9141967; doi:10.3390/ijms23105379)

Supporting information for

DFT study of molecular structure, electronic and vibrational spectra of tetrapyrazinoporphyrazine, its perchlorinated derivative and their Al, Ga and In complexes

Igor V. Ryzhov, Alexey V. Eroshin, Yuriy A. Zhabanov, Daniil N. Finogenov and Pavel A. Stuzhin

Content

Cartesian coordinates of H_2TPyzPA optimized PBE0-D3/def2-TZVP level of theory: 2

Cartesian coordinates of H_2TPyzPACl_8 optimized PBE0-D3/def2-TZVP level of theory: 2

Cartesian coordinates of Al(Cl)TPyzPA optimized PBE0-D3/def2-TZVP level of theory:.............3

Cartesian coordinates of Al(Cl)TPyzPACl_8 optimized PBE0-D3/def2-TZVP level of theory:.........4

Cartesian coordinates of Ga(Cl)TPyzPA optimized PBE0-D3/def2-TZVP level of theory:.............5

Cartesian coordinates of Ga(Cl)TPyzPACl_8 optimized PBE0-D3/def2-TZVP level of theory:........6

Cartesian coordinates of In(Cl)TPyzPA optimized PBE0-D3/def2-TZVP level of theory: 7

Cartesian coordinates of In(Cl)TPyzPACl_8 optimized PBE0-D3/def2-TZVP level of theory: 8

Table S1. Assignment of the IR vibrations of the M(Cl)TPyzPA and M(Cl)TPyzPACl_8 complexes. 9

Figure S1. Shapes of the frontier molecular orbitals. 13

Figure S2. Experimental absorption spectra of Ga(OH)TPyzPACl_8 15

Figure S3. Experimental absorption spectra of In(OH)TPyzPACl_8..........16

Figure S4. Experimental IR spectra of In(OH)TPyzPACl_8..........16

Figure S5. Experimental IR spectra of Ga(OH)TPyzPACl_8 17

Cartesian coordinates of H_2TPyzPA optimized PBE0-D3/def2-TZVP level of theory:

H	-7.316934758	0.000000000	-1.243024521
H	7.316934758	0.000000000	1.243024521
H	-7.316934758	0.000000000	1.243024521
H	7.316934758	0.000000000	-1.243024521
N	0.000000000	0.000000000	1.949373756
N	0.000000000	0.000000000	-1.949373756
N	-2.026802514	0.000000000	0.000000000
N	2.026802514	0.000000000	0.000000000
N	-2.367620345	0.000000000	2.382272070
N	2.367620345	0.000000000	-2.382272070
N	-2.367620345	0.000000000	-2.382272070
N	2.367620345	0.000000000	2.382272070
C	-1.097174988	0.000000000	2.745231468
C	1.097174988	0.000000000	-2.745231468
C	-1.097174988	0.000000000	-2.745231468
C	1.097174988	0.000000000	2.745231468
C	-2.776318572	0.000000000	1.144059492
C	2.776318572	0.000000000	-1.144059492
C	-2.776318572	0.000000000	-1.144059492
C	2.776318572	0.000000000	1.144059492
C	-0.696518733	0.000000000	-4.153144586
C	0.696518733	0.000000000	4.153144586
C	-0.696518733	0.000000000	4.153144586
C	0.696518733	0.000000000	-4.153144586
C	-4.157193213	0.000000000	0.700426626
C	4.157193213	0.000000000	-0.700426626
C	-4.157193213	0.000000000	-0.700426626
C	4.157193213	0.000000000	0.700426626
N	-1.427111726	0.000000000	5.256759227
N	1.427111726	0.000000000	-5.256759227
N	-1.427111726	0.000000000	-5.256759227
N	1.427111726	0.000000000	5.256759227
N	-5.269395784	0.000000000	1.427027058
N	5.269395784	0.000000000	-1.427027058
N	-5.269395784	0.000000000	-1.427027058
N	5.269395784	0.000000000	1.427027058
C	-0.700566260	0.000000000	-6.367646524
C	0.700566260	0.000000000	6.367646524
C	-0.700566260	0.000000000	6.367646524
C	0.700566260	0.000000000	-6.367646524
C	-6.374070875	0.000000000	0.704156276
C	6.374070875	0.000000000	-0.704156276
C	-6.374070875	0.000000000	-0.704156276
C	6.374070875	0.000000000	0.704156276
H	-1.240997007	0.000000000	7.309465400
H	1.240997007	0.000000000	-7.309465400
H	-1.240997007	0.000000000	-7.309465400
H	1.240997007	0.000000000	7.309465400
H	-1.015955264	0.000000000	0.000000000
H	1.015955264	0.000000000	0.000000000

Cartesian coordinates of H_2TPyzPACl_8 optimized PBE0-D3/def2-TZVP level of theory:

Cl	-7.855328018	0.000000000	-1.575057465
Cl	7.855328018	0.000000000	1.575057465
Cl	-7.855328018	0.000000000	1.575057465
Cl	7.855328018	0.000000000	-1.575057465
N	0.000000000	0.000000000	1.946209952
N	0.000000000	0.000000000	-1.946209952

N	-2.025469574	0.000000000	0.000000000
N	2.025469574	0.000000000	0.000000000
N	-2.368401306	0.000000000	2.382389287
N	2.368401306	0.000000000	-2.382389287
N	-2.368401306	0.000000000	-2.382389287
N	2.368401306	0.000000000	2.382389287
C	-1.097557103	0.000000000	2.742346980
C	1.097557103	0.000000000	-2.742346980
C	-1.097557103	0.000000000	-2.742346980
C	1.097557103	0.000000000	2.742346980
C	-2.775881464	0.000000000	1.143976582
C	2.775881464	0.000000000	-1.143976582
C	-2.775881464	0.000000000	-1.143976582
C	2.775881464	0.000000000	1.143976582
C	-0.692826004	0.000000000	-4.146436102
C	0.692826004	0.000000000	4.146436102
C	-0.692826004	0.000000000	4.146436102
C	0.692826004	0.000000000	-4.146436102
C	-4.152681983	0.000000000	0.696982793
C	4.152681983	0.000000000	-0.696982793
C	-4.152681983	0.000000000	-0.696982793
C	4.152681983	0.000000000	0.696982793
N	-1.410985732	0.000000000	5.260212516
N	1.410985732	0.000000000	-5.260212516
N	-1.410985732	0.000000000	-5.260212516
N	1.410985732	0.000000000	5.260212516
N	-5.274259693	0.000000000	1.411367918
N	5.274259693	0.000000000	-1.411367918
N	-5.274259693	0.000000000	-1.411367918
N	5.274259693	0.000000000	1.411367918
C	-0.712073172	0.000000000	-6.368448917
C	0.712073172	0.000000000	6.368448917
C	-0.712073172	0.000000000	6.368448917
C	0.712073172	0.000000000	-6.368448917
C	-6.377530567	0.000000000	0.716524369
C	6.377530567	0.000000000	-0.716524369
C	-6.377530567	0.000000000	-0.716524369
C	6.377530567	0.000000000	0.716524369
Cl	-1.577098249	0.000000000	7.845309081
Cl	1.577098249	0.000000000	-7.845309081
Cl	-1.577098249	0.000000000	-7.845309081
Cl	1.577098249	0.000000000	7.845309081
H	-1.014696494	0.000000000	0.000000000
H	1.014696494	0.000000000	0.000000000

Cartesian coordinates of Al(Cl)TPyzPA optimized PBE0-D3/def2-TZVP level of theory:

H	1.241994419	7.273917468	-0.202632255
H	-7.273917468	1.241994419	-0.202632255
H	-1.241994419	-7.273917468	-0.202632255
H	7.273917468	1.241994419	-0.202632255
H	-1.241994419	7.273917468	-0.202632255
H	-7.273917468	-1.241994419	-0.202632255
H	1.241994419	-7.273917468	-0.202632255
H	7.273917468	-1.241994419	-0.202632255
N	0.000000000	1.929333377	0.009116509
N	-1.929333377	0.000000000	0.009116509
N	0.000000000	-1.929333377	0.009116509
N	1.929333377	0.000000000	0.009116509
N	2.366678363	2.366678363	-0.033707206

N	-2.366678363	2.366678363	-0.033707206
N	-2.366678363	-2.366678363	-0.033707206
N	2.366678363	-2.366678363	-0.033707206
C	1.110944333	2.732166402	-0.028358042
C	-2.732166402	1.110944333	-0.028358042
C	-1.110944333	-2.732166402	-0.028358042
C	2.732166402	1.110944333	-0.028358042
C	-1.110944333	2.732166402	-0.028358042
C	-2.732166402	-1.110944333	-0.028358042
C	1.110944333	-2.732166402	-0.028358042
C	2.732166402	-1.110944333	-0.028358042
C	0.695282268	4.120423776	-0.081054201
C	-4.120423776	0.695282268	-0.081054201
C	-0.695282268	-4.120423776	-0.081054201
C	4.120423776	0.695282268	-0.081054201
C	-0.695282268	4.120423776	-0.081054201
C	-4.120423776	-0.695282268	-0.081054201
C	0.695282268	-4.120423776	-0.081054201
C	4.120423776	-0.695282268	-0.081054201
N	1.429060957	5.226807944	-0.128645129
N	-5.226807944	1.429060957	-0.128645129
N	-1.429060957	-5.226807944	-0.128645129
N	5.226807944	1.429060957	-0.128645129
N	-1.429060957	5.226807944	-0.128645129
N	-5.226807944	-1.429060957	-0.128645129
N	1.429060957	-5.226807944	-0.128645129
N	5.226807944	-1.429060957	-0.128645129
C	0.704144622	6.331100073	-0.167323450
C	-6.331100073	0.704144622	-0.167323450
C	-0.704144622	-6.331100073	-0.167323450
C	6.331100073	0.704144622	-0.167323450
C	-0.704144622	6.331100073	-0.167323450
C	-6.331100073	-0.704144622	-0.167323450
C	0.704144622	-6.331100073	-0.167323450
C	6.331100073	-0.704144622	-0.167323450
Al	0.000000000	0.000000000	0.456662634
Cl	0.000000000	0.000000000	2.601498530

Cartesian coordinates of Al(Cl)TPyzPACl_8 optimized PBE0-D3/def2-TZVP level of theory:

Cl	1.574373732	7.811298732	-0.226583585
Cl	-7.811298732	1.574373732	-0.226583585
Cl	-1.574373732	-7.811298732	-0.226583585
Cl	7.811298732	1.574373732	-0.226583585
Cl	-1.574373732	7.811298732	-0.226583585
Cl	-7.811298732	-1.574373732	-0.226583585
Cl	1.574373732	-7.811298732	-0.226583585
Cl	7.811298732	-1.574373732	-0.226583585
N	0.000000000	1.927877130	0.013273473
N	-1.927877130	0.000000000	0.013273473
N	0.000000000	-1.927877130	0.013273473
N	1.927877130	0.000000000	0.013273473
N	2.367428879	2.367428879	-0.027045342
N	-2.367428879	2.367428879	-0.027045342
N	-2.367428879	-2.367428879	-0.027045342
N	2.367428879	-2.367428879	-0.027045342
C	1.111308267	2.731172476	-0.022629446
C	-2.731172476	1.111308267	-0.022629446
C	-1.111308267	-2.731172476	-0.022629446
C	2.731172476	1.111308267	-0.022629446

C	-1.111308267	2.731172476	-0.022629446
C	-2.731172476	-1.111308267	-0.022629446
C	1.111308267	-2.731172476	-0.022629446
C	2.731172476	-1.111308267	-0.022629446
C	0.691950488	4.115538761	-0.074867860
C	-4.115538761	0.691950488	-0.074867860
C	-0.691950488	-4.115538761	-0.074867860
C	4.115538761	0.691950488	-0.074867860
C	-0.691950488	4.115538761	-0.074867860
C	-4.115538761	-0.691950488	-0.074867860
C	0.691950488	-4.115538761	-0.074867860
C	4.115538761	-0.691950488	-0.074867860
N	1.413415528	5.231421770	-0.124549711
N	-5.231421770	1.413415528	-0.124549711
N	-1.413415528	-5.231421770	-0.124549711
N	5.231421770	1.413415528	-0.124549711
N	-1.413415528	5.231421770	-0.124549711
N	-5.231421770	-1.413415528	-0.124549711
N	1.413415528	-5.231421770	-0.124549711
N	5.231421770	-1.413415528	-0.124549711
C	0.716481190	6.334223103	-0.166294033
C	-6.334223103	0.716481190	-0.166294033
C	-0.716481190	-6.334223103	-0.166294033
C	6.334223103	0.716481190	-0.166294033
C	-0.716481190	6.334223103	-0.166294033
C	-6.334223103	-0.716481190	-0.166294033
C	0.716481190	-6.334223103	-0.166294033
C	6.334223103	-0.716481190	-0.166294033
Al	0.000000000	0.000000000	0.464251706
Cl	0.000000000	0.000000000	2.605926478

Cartesian coordinates of Ga(Cl)TPyzPA optimized PBE0-D3/def2-TZVP level of theory:

H	-6.033090802	4.276647761	0.254999058
H	-4.276647761	-6.033090802	0.254999058
H	6.033090802	-4.276647761	0.254999058
H	4.276647761	-6.033090802	0.254999058
H	6.033090802	4.276647761	0.254999058
H	-4.276647761	6.033090802	0.254999058
H	-6.033090802	-4.276647761	0.254999058
H	4.276647761	6.033090802	0.254999058
N	-1.385115574	1.385115574	-0.010075105
N	-1.385115574	-1.385115574	-0.010075105
N	1.385115574	-1.385115574	-0.010075105
N	1.385115574	1.385115574	-0.010075105
N	-3.353098840	0.000000000	0.040423744
N	0.000000000	-3.353098840	0.040423744
N	3.353098840	0.000000000	0.040423744
N	0.000000000	3.353098840	0.040423744
C	-2.732062581	1.153647513	0.034559390
C	-1.153647513	-2.732062581	0.034559390
C	2.732062581	-1.153647513	0.034559390
C	1.153647513	-2.732062581	0.034559390
C	2.732062581	1.153647513	0.034559390
C	-1.153647513	2.732062581	0.034559390
C	-2.732062581	-1.153647513	0.034559390
C	1.153647513	2.732062581	0.034559390
C	-3.418016544	2.432707548	0.100673428
C	-2.432707548	-3.418016544	0.100673428
C	3.418016544	-2.432707548	0.100673428

C	2.432707548	-3.418016544	0.100673428
C	3.418016544	2.432707548	0.100673428
C	-2.432707548	3.418016544	0.100673428
C	-3.418016544	-2.432707548	0.100673428
C	2.432707548	3.418016544	0.100673428
N	-4.717498531	2.697604607	0.160365284
N	-2.697604607	-4.717498531	0.160365284
N	4.717498531	-2.697604607	0.160365284
N	2.697604607	-4.717498531	0.160365284
N	4.717498531	2.697604607	0.160365284
N	-2.697604607	4.717498531	0.160365284
N	-4.717498531	-2.697604607	0.160365284
N	2.697604607	4.717498531	0.160365284
C	-4.986238030	3.991286150	0.209917920
C	-3.991286150	-4.986238030	0.209917920
C	4.986238030	-3.991286150	0.209917920
C	3.991286150	-4.986238030	0.209917920
C	4.986238030	3.991286150	0.209917920
C	-3.991286150	4.986238030	0.209917920
C	-4.986238030	-3.991286150	0.209917920
C	3.991286150	4.986238030	0.209917920
Ga	0.000000000	0.000000000	-0.509501492
Cl	0.000000000	0.000000000	-2.696011249

Cartesian coordinates of Ga(Cl)TPyzPACl_8 optimized PBE0-D3/def2-TZVP level of theory:

Cl	-6.647807072	4.420506961	0.286875507
Cl	-4.420506961	-6.647807072	0.286875507
Cl	6.647807072	-4.420506961	0.286875507
Cl	4.420506961	-6.647807072	0.286875507
Cl	6.647807072	4.420506961	0.286875507
Cl	-4.420506961	6.647807072	0.286875507
Cl	-6.647807072	-4.420506961	0.286875507
Cl	4.420506961	6.647807072	0.286875507
N	-1.383994533	1.383994533	-0.016747843
N	-1.383994533	-1.383994533	-0.016747843
N	1.383994533	-1.383994533	-0.016747843
N	1.383994533	1.383994533	-0.016747843
N	-3.354069598	0.000000000	0.031295564
N	0.000000000	-3.354069598	0.031295564
N	3.354069598	0.000000000	0.031295564
N	0.000000000	3.354069598	0.031295564
C	-2.731541515	1.152676492	0.026421406
C	-1.152676492	-2.731541515	0.026421406
C	2.731541515	-1.152676492	0.026421406
C	1.152676492	-2.731541515	0.026421406
C	2.731541515	1.152676492	0.026421406
C	-1.152676492	2.731541515	0.026421406
C	-2.731541515	-1.152676492	0.026421406
C	1.152676492	2.731541515	0.026421406
C	-3.412085905	2.431577291	0.092612989
C	-2.431577291	-3.412085905	0.092612989
C	3.412085905	-2.431577291	0.092612989
C	2.431577291	-3.412085905	0.092612989
C	3.412085905	2.431577291	0.092612989
C	-2.431577291	3.412085905	0.092612989
C	-3.412085905	-2.431577291	0.092612989
C	2.431577291	3.412085905	0.092612989
N	-4.709552792	2.711946695	0.155618140
N	-2.711946695	-4.709552792	0.155618140

N	4.709552792	-2.711946695	0.155618140
N	2.711946695	-4.709552792	0.155618140
N	4.709552792	2.711946695	0.155618140
N	-2.711946695	4.709552792	0.155618140
N	-4.709552792	-2.711946695	0.155618140
N	2.711946695	4.709552792	0.155618140
C	-4.996931635	3.984618302	0.209302700
C	-3.984618302	-4.996931635	0.209302700
C	4.996931635	-3.984618302	0.209302700
C	3.984618302	-4.996931635	0.209302700
C	4.996931635	3.984618302	0.209302700
C	-3.984618302	4.996931635	0.209302700
C	-4.996931635	-3.984618302	0.209302700
C	3.984618302	4.996931635	0.209302700
Ga	0.000000000	0.000000000	-0.521137775
Cl	0.000000000	0.000000000	-2.703696590

Cartesian coordinates of In(Cl)TPyzPA optimized PBE0-D3/def2-TZVP level of theory:

N	-1.427128697	-1.427128697	-0.184954410
N	1.427128697	-1.427128697	-0.184954410
N	1.427128697	1.427128697	-0.184954410
N	-1.427128697	1.427128697	-0.184954410
N	0.000000000	-3.362769055	-0.042176186
N	3.362769055	0.000000000	-0.042176186
N	0.000000000	3.362769055	-0.042176186
N	-3.362769055	0.000000000	-0.042176186
C	-1.170218007	-2.760917501	-0.064946988
C	2.760917501	-1.170218007	-0.064946988
C	1.170218007	2.760917501	-0.064946988
C	-2.760917501	-1.170218007	-0.064946988
C	1.170218007	-2.760917501	-0.064946988
C	2.760917501	1.170218007	-0.064946988
C	-1.170218007	2.760917501	-0.064946988
C	-2.760917501	1.170218007	-0.064946988
C	-2.449449924	-3.439028117	0.105287075
C	3.439028117	-2.449449924	0.105287075
C	2.449449924	3.439028117	0.105287075
C	-3.439028117	-2.449449924	0.105287075
C	2.449449924	-3.439028117	0.105287075
C	3.439028117	2.449449924	0.105287075
C	-2.449449924	3.439028117	0.105287075
C	-3.439028117	2.449449924	0.105287075
N	-2.711859056	-4.729769116	0.260049776
N	4.729769116	-2.711859056	0.260049776
N	2.711859056	4.729769116	0.260049776
N	-4.729769116	-2.711859056	0.260049776
N	2.711859056	-4.729769116	0.260049776
N	4.729769116	2.711859056	0.260049776
N	-2.711859056	4.729769116	0.260049776
N	-4.729769116	2.711859056	0.260049776
C	-4.000819904	-4.994439790	0.399470887
C	4.994439790	-4.000819904	0.399470887
C	4.000819904	4.994439790	0.399470887
C	-4.994439790	-4.000819904	0.399470887
C	4.000819904	-4.994439790	0.399470887
C	4.994439790	4.000819904	0.399470887
C	-4.000819904	4.994439790	0.399470887
C	-4.994439790	4.000819904	0.399470887
In	0.000000000	0.000000000	-0.957445728

Cl	0.000000000	0.000000000	-3.293544009
H	-4.279901123	-6.036546460	0.523348892
H	6.036546460	-4.279901123	0.523348892
H	4.279901123	6.036546460	0.523348892
H	-6.036546460	-4.279901123	0.523348892
H	4.279901123	-6.036546460	0.523348892
H	6.036546460	4.279901123	0.523348892
H	-4.279901123	6.036546460	0.523348892
H	-6.036546460	4.279901123	0.523348892

Cartesian coordinates of In(Cl)TPyzPACl$_8$ optimized PBE0-D3/def2-TZVP level of theory:

Cl	4.419820852	6.648463481	0.598162105
Cl	-6.648463481	4.419820852	0.598162105
Cl	-4.419820852	-6.648463481	0.598162105
Cl	6.648463481	4.419820852	0.598162105
Cl	-4.419820852	6.648463481	0.598162105
Cl	-6.648463481	-4.419820852	0.598162105
Cl	4.419820852	-6.648463481	0.598162105
Cl	6.648463481	-4.419820852	0.598162105
N	1.425210180	1.425210180	-0.183437170
N	-1.425210180	1.425210180	-0.183437170
N	-1.425210180	-1.425210180	-0.183437170
N	1.425210180	-1.425210180	-0.183437170
N	0.000000000	3.363559137	-0.042309459
N	-3.363559137	0.000000000	-0.042309459
N	0.000000000	-3.363559137	-0.042309459
N	3.363559137	0.000000000	-0.042309459
C	1.169016335	2.759830412	-0.064921815
C	-2.759830412	1.169016335	-0.064921815
C	-1.169016335	-2.759830412	-0.064921815
C	2.759830412	1.169016335	-0.064921815
C	-1.169016335	2.759830412	-0.064921815
C	-2.759830412	-1.169016335	-0.064921815
C	1.169016335	-2.759830412	-0.064921815
C	2.759830412	-1.169016335	-0.064921815
C	2.448195495	3.432718213	0.103302984
C	-3.432718213	2.448195495	0.103302984
C	-2.448195495	-3.432718213	0.103302984
C	3.432718213	2.448195495	0.103302984
C	-2.448195495	3.432718213	0.103302984
C	-3.432718213	-2.448195495	0.103302984
C	2.448195495	-3.432718213	0.103302984
C	3.432718213	-2.448195495	0.103302984
N	2.726146361	4.721437025	0.260533022
N	-4.721437025	2.726146361	0.260533022
N	-2.726146361	-4.721437025	0.260533022
N	4.721437025	2.726146361	0.260533022
N	-2.726146361	4.721437025	0.260533022
N	-4.721437025	-2.726146361	0.260533022
N	2.726146361	-4.721437025	0.260533022
N	4.721437025	-2.726146361	0.260533022
C	3.993873612	5.004692168	0.401660717
C	-5.004692168	3.993873612	0.401660717
C	-3.993873612	-5.004692168	0.401660717
C	5.004692168	3.993873612	0.401660717
C	-3.993873612	5.004692168	0.401660717
C	-5.004692168	-3.993873612	0.401660717
C	3.993873612	-5.004692168	0.401660717
C	5.004692168	-3.993873612	0.401660717

```
In        0.000000000       0.000000000      -0.965648517
Cl        0.000000000       0.000000000      -3.297200007
```

Table S1. Assignment of the IR vibrations of the M(Cl)TPyzPA and M(Cl)TPyzPACl$_8$ complexes.

Frequency, cm^{-1}	I_{rel}, %	Symmetry	Assignment[1]	Exp, cm^{-1}
**H_2TPyzPA**				
655 (ω_{46})	32	B_{1u}	r(N_p-C_α) (8), r(N_m-C_α) (21), φ(C_α-N_p-C_α) (7), φ(N_p-C_α-C_β) (9), φ(C_β-C_β-N_d) (11), φ(N_d-C_γ-C_γ) (11)	
745 (ω_{54})	29	B_{2u}	r(C_α-C_β) (14), r(C_β-N_d) (16), φ(C_α-N_p-C_α) (5), φ(N_p-C_α-C_β) (8), φ(N_p-C_α-N_m) (6), φ(C_β-N_d-C_γ) (14)	
810 (ω_{62})	28	B_{3u}	OPB(H_c-C_α-C_α-N_p) (39), OPB (C_β-N_p-N_p-C_α) (25), θ(C_α-H_c-N_p-C_α-N_m-C_β) (24)	
1055 (ω_{81})	53	B_{1u}	r(N_p-C_α) (25), r(N_m-C_α) (22), r(C_α-C_β) (7), r(C_γ-C_γ) (20)	
1091 (ω_{86})	78	B_{1u}	r(N_p-C_α) (29), r(N_m-C_α) (18), r(N_d-C_γ) (10), r(C_γ-C_γ) (12)	
1157 (ω_{90})	81	B_{2u}	r(N_p-C_α) (23), r(N_m-C_α) (18), r(C_α-C_β) (6), r(C_β-N_d) (8), r(N_d-C_γ) (6), φ(C_α-N_p-H_c) (8), φ(C_β-N_d-C_γ) (7)	
1216 (ω_{92})	57	B_{2u}	r(N_p-C_α) (12), r(C_β-C_β) (9), r(N_d-C_γ) (25), r(C_γ-C_γ) (7), φ(C_α-N_p-H_c) (10)	
1242 (ω_{95})	100	B_{1u}	r(C_α-C_β) (14), r(C_β-C_β) (10), r(N_d-C_γ) (10), φ(N_d-C_γ-H_s) (7) r(C_β-N_d) (6), r(C_β-C_β) (42), r(C_γ-Cl) (26), φ(N_d-C_γ-Cl) (6)	
1400 (ω_{111})	22	B_{1u}	r(N_m-C_α) (9), r(C_β-C_β) (6), r(C_γ-C_γ) (8), φ(N_d-C_γ-H_s) (30), φ(C_γ-C_γ-H_s) (19)	
1406 (ω_{113})	37	B_{2u}	r(C_β-C_β) (12), r(C_γ-C_γ) (13), φ(N_d-C_γ-H_s) (31), φ(C_γ-C_γ-H_s) (20)	
1583 (ω_{121})	38	B_{1u}	r(N_p-C_α) (11), r(N_m-C_α) (63), r(C_α-C_β) (8)	
1583 (ω_{122})	16	B_{2u}	r(N_p-C_α) (10), r(N_m-C_α) (54), r(C_α-C_β) (8)	
3193 (ω_{139})	20	B_{1u}	r(H_s-C_γ) (99)	
3193 (ω_{141})	24	B_{2u}	r(H_s-C_γ) (99)	
3552 (ω_{143})	45	B_{1u}	r(H_c-N_p) (99)	
**H_2TPyzPACl$_8$**				
692 (ω_{70})	31	B_{1u}	r(N_p-C_α) (9), r(N_m-C_α) (23), r(C_γ-Cl) (10), φ(C_α-N_p-C_α) (6), φ(C_α-C_β-C_β) (7), φ(C_α-N_p-C_α) (8)	
769 (ω_{79})	48	B_{2u}	r(C_α-C_β) (17), r(C_γ-Cl) (14), r(C_β-N_d) (8), φ(N_p-C_α-C_β) (6), φ(N_p-C_α-N_m) (10), φ(N_p-C_α-C_β) (7)	
786(ω_{82})	30	B_{1u}	r(C_α-C_β) (21), r(C_β-N_d) (10), r(C_γ-Cl) (20), φ(N_p-C_α-N_m) (10), φ(C_β-C_β-N_d) (6)	
998 (ω_{95})	40	B_{1u}	r(N_m-C_α) (14), r(C_α-C_β) (10), r(C_γ-Cl) (36), φ(N_d-C_γ-C_γ) (17)	
1080 (ω_{97})	45	B_{1u}	r(N_p-C_α) (43), r(N_m-C_α) (31), r(C_α-C_β) (5)	
1108 (ω_{99})	30	B_{2u}	r(N_p-C_α) (20), r(C_γ-Cl) (13), φ(C_α-N_p-H_c) (10), φ(C_α-C_β-N_d) (7), φ(C_β-N_d-C_γ) (16),	

			$\varphi(C_\beta$-C_β-$N_d)$ (11)
1176 (ω_{102})	33	B_{2u}	r(N_p-C_α) (19), r(N_m-C_α) (21), r(C_α-C_β) (7), r(C_β-C_β) (6), r(C_β-N_d) (8), φ(C_α-N_p-H_c) (10), φ(C_β-N_d-C_γ) (8)
1242 (ω_{107})	100	B_{1u}	r(C_β-N_d) (6), r(C_β-C_β) (42), r(C_γ-Cl) (26), φ(N_d-C_γ-Cl) (6)
1255 (ω_{109})	98	B_{2u}	r(C_β-N_d) (8), r(C_γ-C_γ) (46), r(C_γ-Cl) (21), φ(N_d-C_γ-Cl) (6)
1281 (ω_{111})	67	B_{1u}	r(C_α-C_β) (8), r(C_β-C_β) (19), r(N_d-C_γ) (13), r(C_γ-C_γ) (13)
1315 (ω_{114})	48	B_{2u}	r(N_p-C_α) (5), r(C_β-N_d) (14), r(N_d-C_γ) (55), φ(C_α-N_p-H_c) (8)
1328 (ω_{116})	52	B_{1u}	r(N_p-C_α) (7), r(N_m-C_α) (11), r(C_β-N_d) (12), r(N_d-C_γ) (45)
1340 (ω_{117})	83	B_{2u}	r(N_p-C_α) (8), r(C_α-C_β) (10), r(C_β-C_β) (8), r(C_β-N_d) (19), φ(C_α-N_p-H_c) (28)
1379(ω_{120})	22	B_{1u}	r(N_p-C_α) (20), r(N_m-C_α) (13), r(C_α-C_β) (5), r(C_β-N_d) (12), r(N_d-C_γ) (21), φ(N_p-C_α-N_m) (7)
3554 (ω_{143})	27	B_{1u}	r(H_c-N_p) (99)
**Al(Cl)TPyzPA**			
513 (ω_{40})	14	A_1	r(Al-Cl) (75), φ(N_p-Al-N_p) (5)
943(ω_{78}-ω_{79})	29	E	r(N_m-C_α) (11), r(C_α-C_β) (9), φ(C_α-N_m-C_α) (11), φ(N_p-C_α-N_m) (10), φ(N_m-C_α-C_β) (6), φ(N_d-C_γ-C_γ) (18)
1140 (ω_{92}-ω_{93})	53	E	r(N_p-C_α) (24), r(N_m-C_α) (14), r(C_α-C_β) (10), r(C_β-N_d) (12), r(N_d-C_γ) (9)
1255 (ω_{98}-ω_{99})	100	E	r(N_p-C_α) (5), r(C_α-C_β) (15), r(C_β-C_β) (14), r(N_d-C_γ) (20), φ(C_β-N_d-C_γ) (9)
1289 (ω_{101}-ω_{102})	18	E	r(C_β-N_d) (20), r(N_d-C_γ) (51), r(C_γ-C_γ) (7)
1404(ω_{113}-ω_{114})	36	E	r(C_β-C_β) (12), r(C_γ-C_γ) (13), φ(N_d-C_γ-H_s) (36), φ(C_γ-C_γ-H_s) (23)
1596 (ω_{125}-ω_{126})	24	E	r(N_p-C_α) (6), r(N_m-C_α) (60), r(C_α-C_β) (8)
3189 (ω_{142}-ω_{143})	18	E	r(H_s-N_p) (99)
**Al(Cl)TPyzPACl$_8$**			
813 (ω_{86}-ω_{87})	36	E	r(N_p-Al) (8), r(C_α-C_β) (7), r(C_β-C_β) (7), r(C_γ-Cl) (15), φ(N_p-C_α-N_m) (7), φ(N_m-C_α-C_β) (9)
1016 (ω_{97}-ω_{98})	40	E	r(N_m-C_α) (6), r(C_α-C_β) (8), r(C_γ-Cl) (38), φ(C_β-N_d-C_γ) (5), φ(C_β-C_β-N_d) (19)
1246 (ω_{111}-ω_{112})	100	E	r(C_γ-C_γ) (43), r(C_γ-Cl) (25), φ(N_d-C_γ-Cl) (6)
1293 (ω_{115}-ω_{116})	52	E	r(N_p-C_α) (8), r(C_β-C_β) (15), r(N_d-C_γ) (25), r(C_γ-C_γ) (13)
1331 (ω_{117}-ω_{118})	97	E	r(C_α-C_β) (8), r(C_β-N_d) (23), r(N_d-C_γ) (49)
1619 (ω_{140}-ω_{141})	15	E	r(N_m-C_α) (6), r(C_α-C_β) (13), r(C_β-N_d) (45), r(N_d-C_γ) (17)
**Ga(Cl)TPyzPA**			
781(ω_{61}-ω_{62})	18	E	r(N_p-Ga) (7), r(C_α-C_β) (8), r(C_β-N_d) (7), φ(N_p-C_α-N_m) (5), φ(N_m-C_α-C_β) (6),

			OPB(C_β-N_p-N_p-C_α) (15)	
938 (ω_{78}-ω_{79})	27	E	r(N_m-C_α) (12), r(C_α-C_β) (8), φ(C_α-N_m-C_α) (12), φ(N_p-C_α-N_m) (10), φ(N_m-C_α-C_β) (8), φ(N_d-C_γ-C_γ) (18)	
1142 (ω_{92}-ω_{93})	44	E	r(N_p-C_α) (49), r(N_m-C_α) (11), r(C_α-C_β) (9), r(N_d-C_γ) (9)	
1254 (ω_{98}-ω_{99})	100	E	r(N_p-C_α) (6), r(C_α-C_β) (12), r(C_β-C_β) (11), r(C_β-N_d) (9), r(N_d-C_γ) (18)	
1288 (ω_{101}-ω_{102})	16	E	r(N_m-C_α) (5), r(C_β-N_d) (20), r(N_d-C_γ) (46), r(C_γ-C_γ) (7)	
1401 (ω_{113}-ω_{114})	35	E	r(C_β-C_β) (11), r(C_γ-C_γ) (12), φ(N_d-C_γ-H_s) (36), φ(C_γ-C_γ-H_s) (23)	
1586 (ω_{125}-ω_{126})	22	E	r(N_p-C_α) (7), r(N_m-C_α) (60), r(C_α-C_β) (9)	
3194 (ω_{142}-ω_{143})	17	E	r(H_s-N_p) (99)	
**Ga(Cl)TPyzPACl$_8$**				
812 (ω_{86}-ω_{87})	33	E	r(N_p-Ga) (6), r(C_α-C_β) (14), r(C_β-N_d) (8), r(C_γ-Cl) (16), φ(N_p-C_α-N_m) (10), φ(N_m-C_α-C_β) (10), φ(C_β-C_β-N_d) (6)	850
877 (ω_{94}-ω_{95})	15	E	r(N_p-Ga) (7), r(N_m-C_α) (5), r(N_m-C_α) (5), r(C_α-C_β) (9), r(C_β-N_d) (9), r(C_γ-Cl) (11), φ(N_p-C_α-N_m) (6), φ(C_α-N_m-C_α) (15), φ(N_m-C_α-C_β) (12)	885
1013 (ω_{97}-ω_{98})	39	E	r(N_m-C_α) (7), r(C_α-C_β) (9), r(C_γ-Cl) (38), φ(N_d-C_γ-C_γ) (19)	
1155 (ω_{104}-ω_{105})	17	E	r(N_p-C_α) (28), r(N_m-C_α) (16), r(C_α-C_β) (11), r(C_β-N_d) (11), φ(C_β-N_d-C_γ) (8)	
1248 (ω_{111}-ω_{112})	100	E	r(C_γ-C_γ) (41), r(C_γ-Cl) (24), φ(N_d-C_γ-Cl) (6)	1232
1290 (ω_{115}-ω_{116})	50	E	r(N_p-C_α) (6), r(C_α-C_β) (7), r(C_β-C_β) (17), r(N_d-C_γ) (26), r(C_γ-C_γ) (13)	
1328 (ω_{117}-ω_{118})	94	E	r(C_α-C_β) (8), r(C_β-N_d) (22), r(N_d-C_γ) (50)	1349
1379 (ω_{121}-ω_{122})	16	E	r(N_p-C_α) (24), r(N_m-C_α) (9), r(C_α-C_β) (14), r(N_d-C_γ) (11)	1401
1619 (ω_{141}-ω_{142})	14	E	r(C_α-C_β) (10), r(C_β-N_d) (48), r(N_d-C_γ) (20), φ(C_β-C_β-N_d) (6)	1687
**In(Cl)TPyzPA**				
778 (ω_{61}-ω_{62})	21	E	r(N_p-In) (6), r(C_α-C_β) (8), r(C_β-N_d) (7), φ(N_p-C_α-N_m) (5), φ(N_m-C_α-C_β) (13), OPB(C_β-N_p-N_p-C_α) (8)	
927 (ω_{78}-ω_{79})	28	E	r(N_m-C_α) (13), r(C_α-C_β) (11), φ(C_α-N_m-C_α) (11), φ(N_p-C_α-N_m) (10), φ(N_m-C_α-C_β) (6), φ(N_d-C_γ-C_γ) (16)	
1143 (ω_{92}-ω_{93})	36	E	r(N_p-C_α) (49), r(N_m-C_α) (10), r(C_α-C_β) (6), r(N_d-C_γ) (10)	
1247 (ω_{98}-ω_{99})	100	E	r(N_p-C_α) (5), r(C_α-C_β) (15), r(C_β-C_β) (14), r(N_d-C_γ) (20)	
1396 (ω_{113}-ω_{114})	34	E	r(C_β-C_β) (10), r(C_γ-C_γ) (10), φ(N_d-C_γ-H_s) (34), φ(C_γ-C_γ-H_s) (22)	
1558 (ω_{124}-ω_{125})	24	E	r(N_p-C_α) (9), r(N_m-C_α) (60), r(C_α-C_β) (10)	
3194 (ω_{142}-ω_{143})	17	E	r(H_s-N_p) (99)	

**In(Cl)TPyzPACl$_8$**				
807 (ω_{86}-ω_{87})	30	E	r(C_α-C_β) (13), r(C_β-N_d) (5), r(C_γ-Cl) (13), φ(N_p-C_α-N_m) (11), φ(N_m-C_α-C_β) (12)	
871 (ω_{94}-ω_{95})	19	E	r(N_p-In) (8), r(N_m-C_α) (6), r(C_α-C_β) (10), r(C_β-N_d) (9), r(C_γ-Cl) (11), φ(N_p-C_α-N_m) (5), φ(C_α-N_m-C_α) (15), φ(N_m-C_α-C_β) (12)	
1005 (ω_{97}-ω_{98})	40	E	r(N_m-C_α) (8), r(C_α-C_β) (10), r(C_γ-Cl) (38), φ(C_β-C_β-N_d) (18)	
1151 (ω_{104}-ω_{105})	18	E	r(N_p-C_α) (41), r(N_m-C_α) (15), r(C_α-C_β) (10), r(C_β-N_d) (8)	
1250 (ω_{111}-ω_{112})	100	E	r(C_γ-C_γ) (44), r(C_γ-Cl) (26), φ(N_d-C_γ-Cl) (6)	1264
1280 (ω_{114}-ω_{115})	50	E	r(N_p-C_α) (5), r(C_α-C_β) (7), r(C_β-C_β) (19), r(N_d-C_γ) (26)	1323
1325 (ω_{117}-ω_{118})	83	E	r(C_α-C_β) (8), r(C_β-N_d) (23), r(N_d-C_γ) (49)	1364
1372 (ω_{121}-ω_{122})	31	E	r(N_m-C_α) (6), r(N_p-C_α) (9), r(C_β-N_d) (20), r(N_d-C_γ) (46), r(C_γ-C_γ) (12)	1507
1617 (ω_{140}-ω_{141})	12	E	r(C_α-C_β) (9), r(C_β-N_d) (51), r(N_d-C_γ) (20)	1701

Figure S1. Shapes of the frontier molecular orbitals.

Al(Cl)TPyzPA

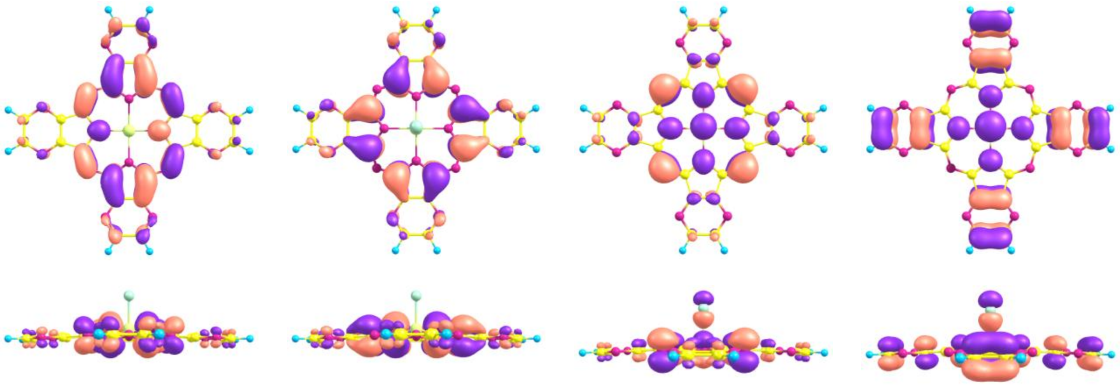

$1e^*$ $3a_2$ $4a_1$ $3a_1$

Ga(Cl)TPyzPA

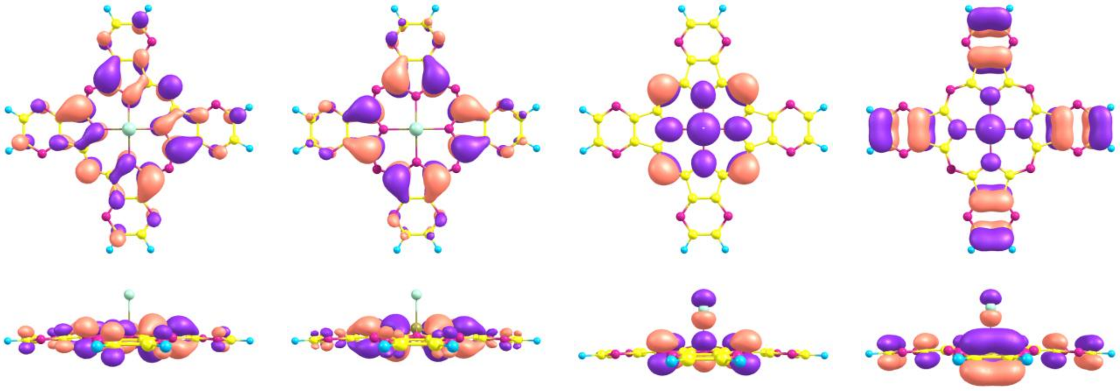

$1e^*$ $3a_2$ $4a_1$ $3a_1$

In(Cl)TPyzPA

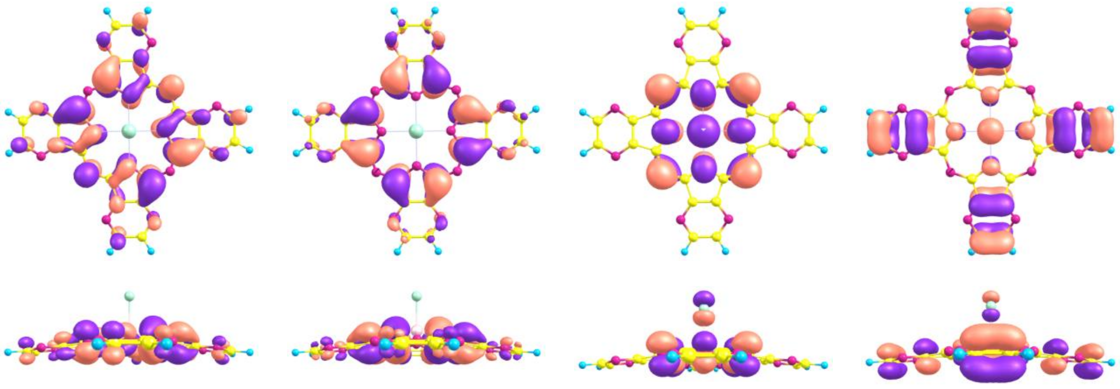

$1e^*$ $3a_2$ $4a_1$ $3a_1$

H_2TPyzPA

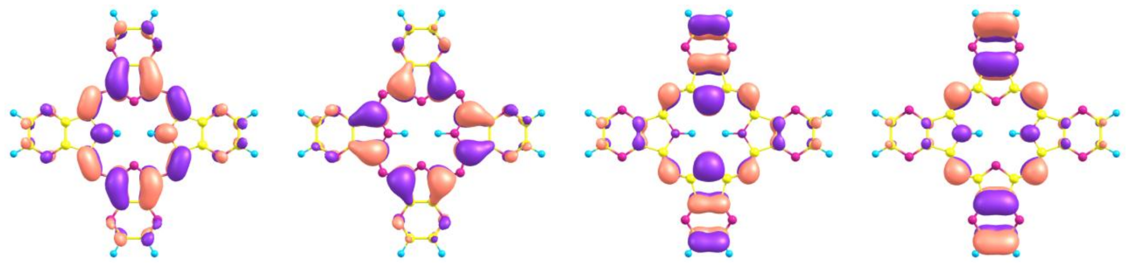

$1b_{1g}^{*}$ $3a_{u}$ $3b_{2u}$ $2b_{2u}$

Al(Cl)TPyzPACl$_8$

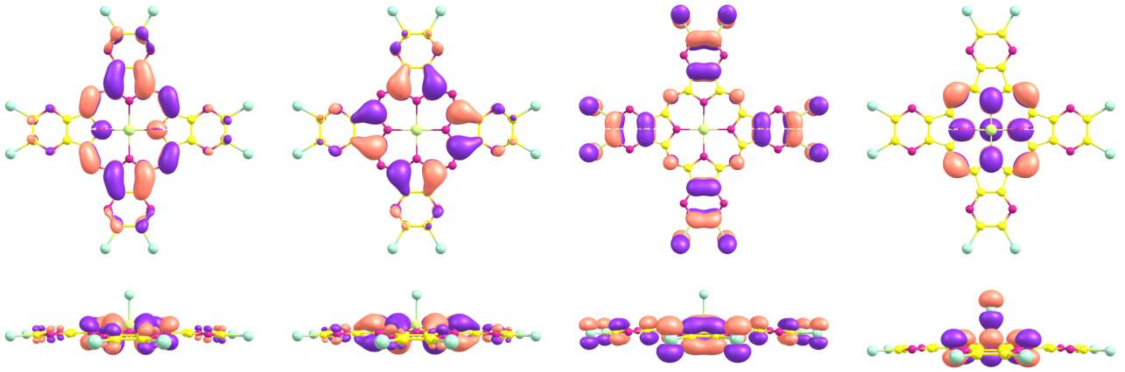

$1e^{*}$ $3a_{2}$ $6a_{1}$ $5a_{1}$

Ga(Cl)TPyzPACl$_8$

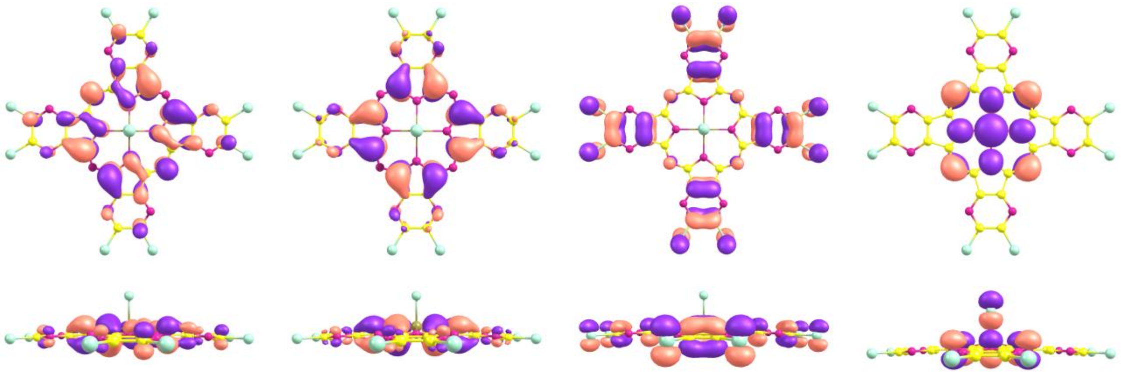

$1e^{*}$ $3a_{2}$ $6a_{1}$ $5a_{1}$

In(Cl)TPyzPACl$_8$

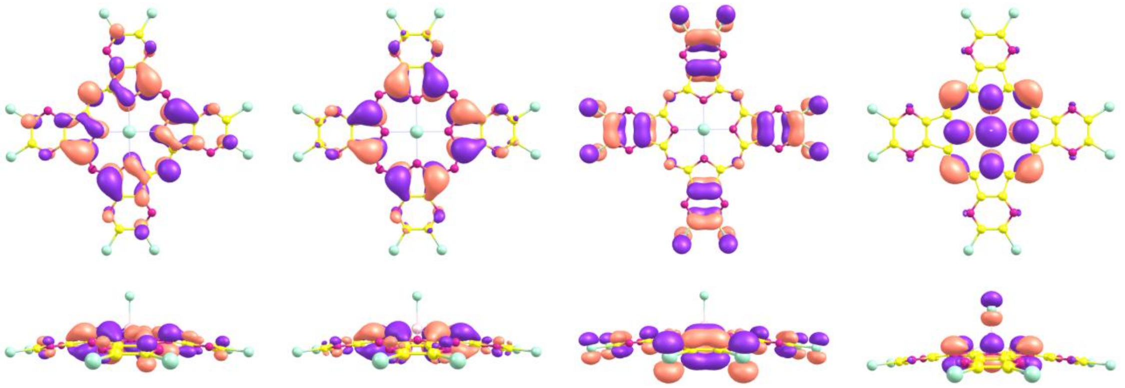

$1e^{*}$ $3a_{2}$ $6a_{1}$ $5a_{1}$

$H_2TPyzPACl_8$

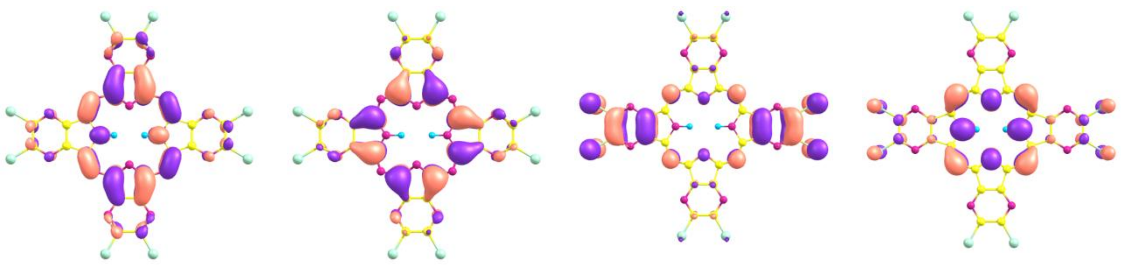

$1b_{1g}^*$ $3a_u$ $1b_{2u}$ $2b_{2u}$

Figure S2. Experimental absorption spectra of $Ga(OH)TPyzPACl_8$

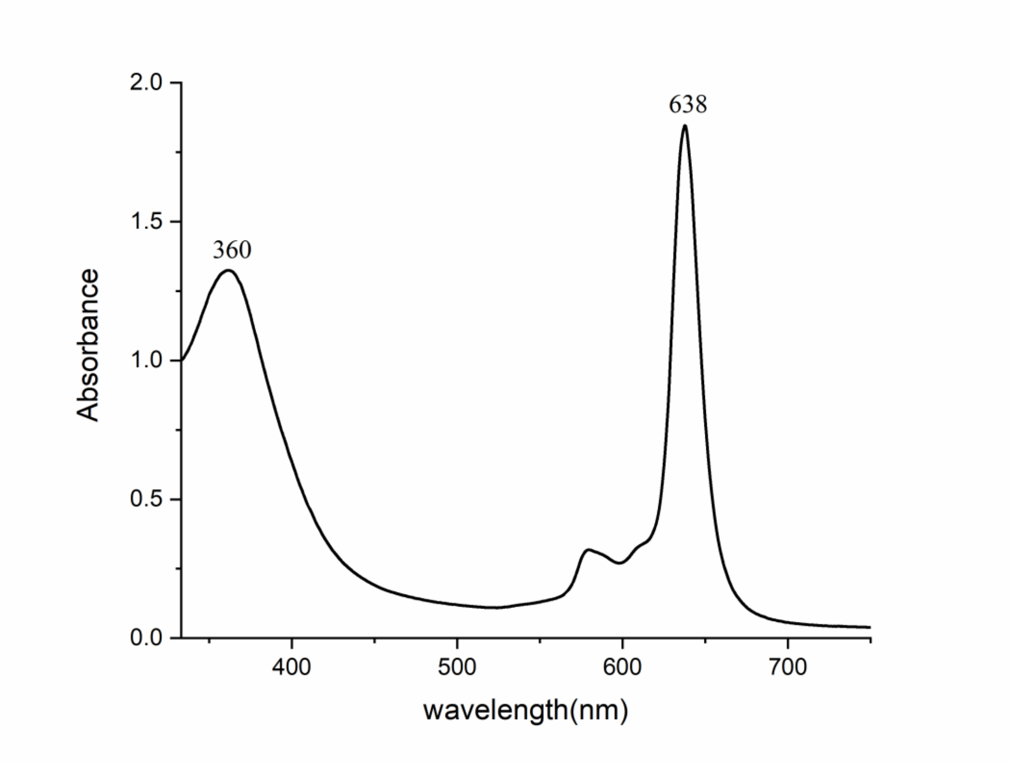

Figure S3. Experimental absorption spectra of In(OH)TPyzPACl8

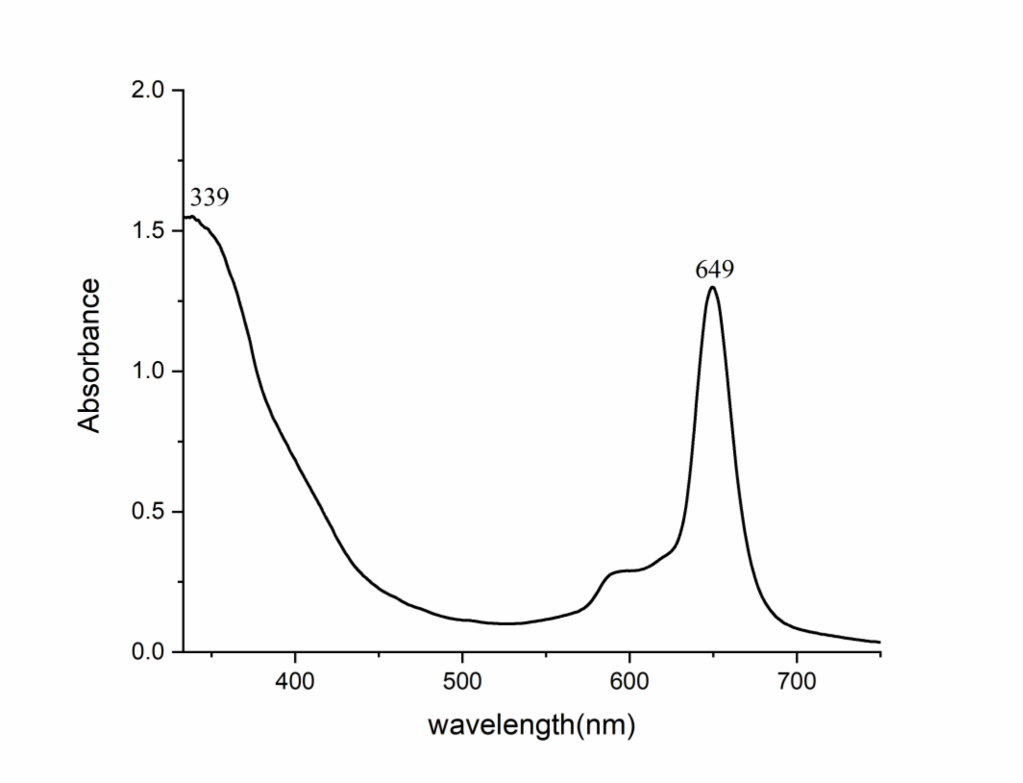

Figure S4. Experimental IR spectra of In(OH)TPyzPACl8

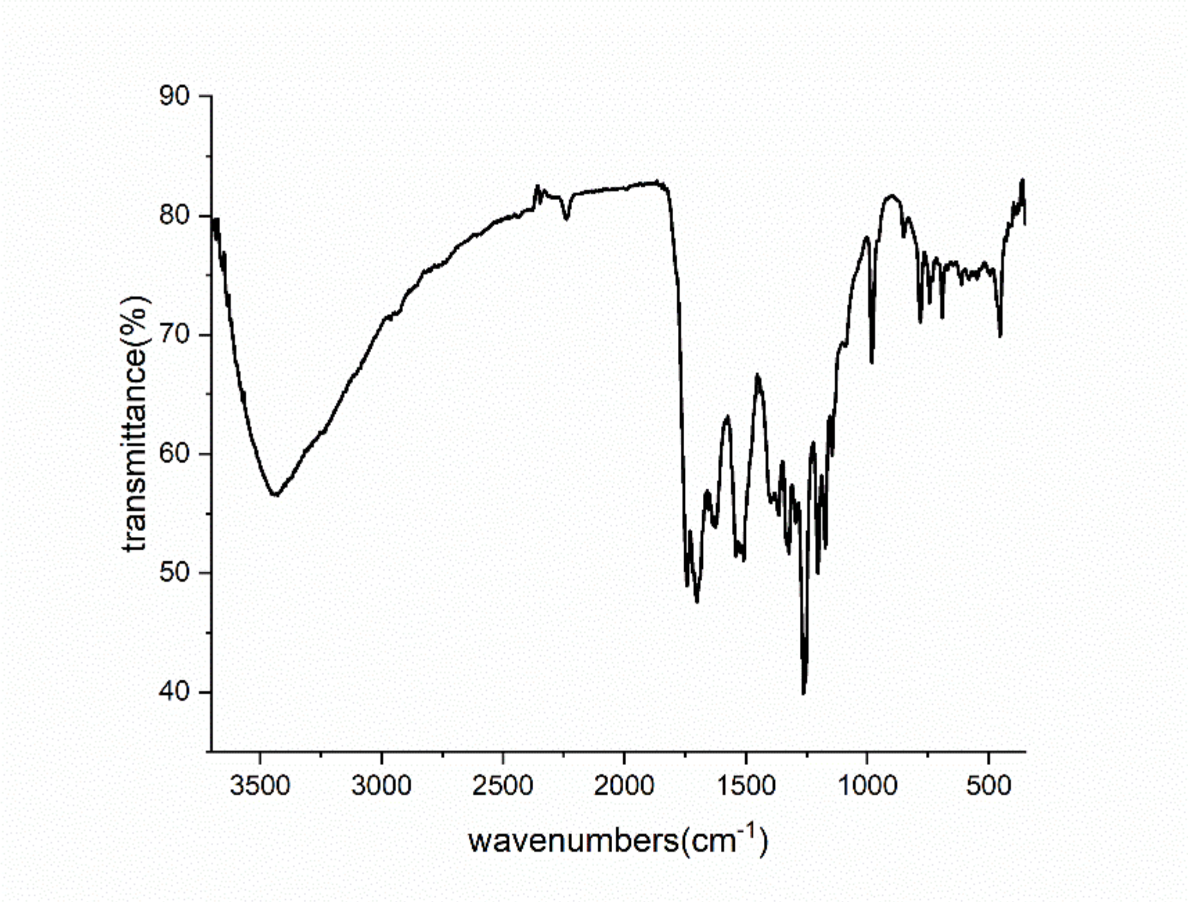

Figure S5. Experimental IR spectra of Ga(OH)TPyzPACl8

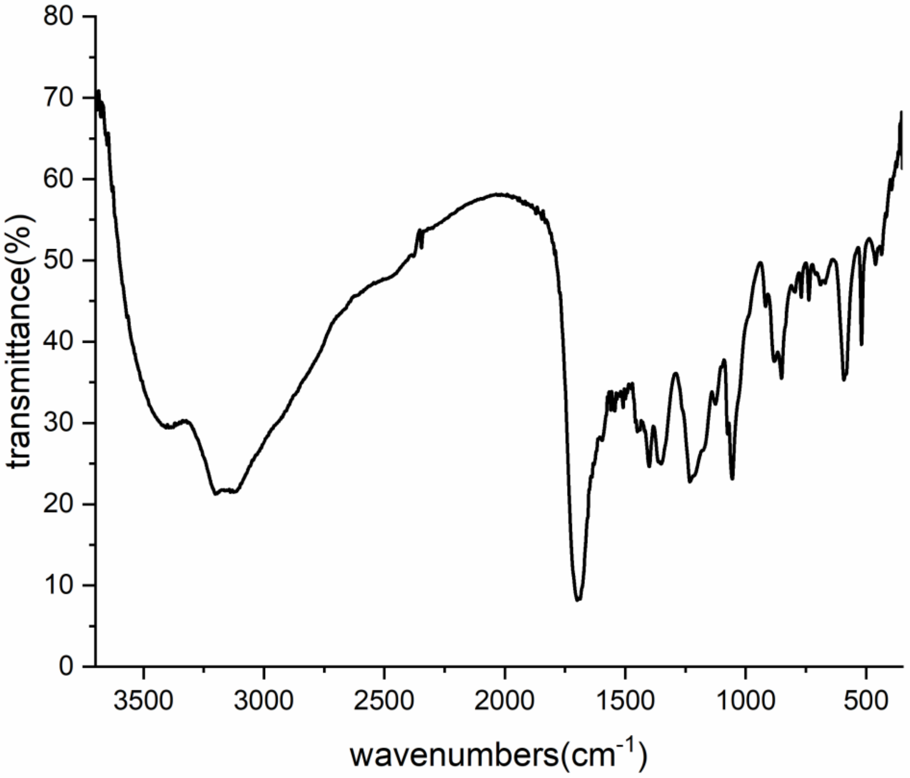

Supplement: Supplementary file 1 [file ijms-23-05379-s001.zip › ijms-1710478-supplementary.pdf]
